# A Comprehensive Phenotypic Investigation of the “Pod-Shattering Syndrome” in Common Bean

**DOI:** 10.3389/fpls.2017.00251

**Published:** 2017-03-03

**Authors:** Maria L. Murgia, Giovanna Attene, Monica Rodriguez, Elena Bitocchi, Elisa Bellucci, Davide Fois, Laura Nanni, Tania Gioia, Diego M. Albani, Roberto Papa, Domenico Rau

**Affiliations:** ^1^Dipartimento di Agraria, Sezione di Agronomia, Colture Erbacee e Genetica, Università degli Studi di SassariSassari, Italy; ^2^Dipartimento di Scienze Agrarie, Alimentari ed Ambientali, Università Politecnica delle MarcheAncona, Italy; ^3^Scuola di Scienze Agrarie, Alimentari, Forestali ed Ambientali, Università degli Studi della BasilicataPotenza, Italy; ^4^Dipartimento di Agraria, Sezione di Economia e Sistemi Arborei e Forestali, Università degli Studi di SassariSassari, Italy

**Keywords:** domestication, domestication syndrome, shattering, common bean, phenotypic analysis, element composition analysis, cell wall analysis

## Abstract

Seed shattering in crops is a key domestication trait due to its relevance for seed dispersal, yield, and fundamental questions in evolution (e.g., convergent evolution). Here, we focused on pod shattering in common bean (*Phaseolus vulgaris* L.), the most important legume crop for human consuption in the world. With this main aim, we developed a methodological pipeline that comprises a thorough characterization under field conditions, including also the chemical composition and histological analysis of the pod valves. The pipeline was developed based on the assumption that the shattering trait itself can be treated in principle as a “syndrome” (i.e., a set of correlated different traits) at the pod level. We characterized a population of 267 introgression lines that were developed *ad-hoc* to study shattering in common bean. Three main objectives were sought: (1) to dissect the shattering trait into its “components,” of *level* (percentage of shattering pods per plant) and *mode* (percentage of pods with twisting or non-twisting valves); (2) to test whether shattering is associated to the chemical composition and/or the histological characteristics of the pod valves; and (3) to test the associations between shattering and other plant traits. We can conclude the following: Very high shattering levels can be achieved in different modes; shattering resistance is mainly a qualitative trait; and high shattering levels is correlated with high carbon and lignin contents of the pod valves and with specific histological charaterstics of the ventral sheath and the inner fibrous layer of the pod wall. Our data also suggest that shattering comes with a “cost,” as it is associated with low pod size, low seed weight per pod, high pod weight, and low seed to pod-valves ratio; indeed, it can be more exaustively described as a syndrome at the pod level. Our work suggests that the valve chemical composition (i.e., carbon and lignin content) can be used for a high troughput phenotyping procedures for shattering phenotyping. Finally, we believe that the application of our pipeline will greatly facilitate comparative studies among legume crops, and gene tagging.

## Introduction

The loss of seed shattering occurred independently in several crops and in different areas of the world during the domestication of many food crops, as this loss was crucial for adaptation of the plants to the agro-ecosystem, to provide ancient farmers with easier and more abundant harvests (Tang et al., 2013). Non-shattering/indehiscent types emerged in maize, barley, and rice (see Li and Olsen, 2016, for a review). Maize was domesticated in the New World, in Mexico, while barley and rice were domesticated in the Fertile Crescent of the Old World and in south-east Asia, respectively. Similarly, among the legume crops, indehiscent phenotypes emerged in soybean and common bean, which were domesticated in the Old World and the New World, respectively (Hymowitz, 1970; Harlan, 1992; Bitocchi et al., 2012; Schmutz et al., 2014). However, fully indehiscent phenotype emerged in common bean only after domestication with the development of snap varieties that are used for the production of green beans due to the absence of fiber strings along the pod valves. In other domesticated commercial classes (e.g., dry beans) shattering traits it is only reduced from that observable in wild populations.

Thus, deciphering the genetic basis of pod shattering is important for evolutionary studies, particularly to unravel the mechanisms of parallel evolution (Lin et al., 2012; Dong and Wang, 2015), and also because this will provide breeders with key information to manipulate this trait to reduce yield loss (Singh, 2001; Santalla et al., 2004). For the same reason, the genomic information would be a great tool to facilitate the exploitation of exotic germplasm in common bean breeding. The potential of these studies is well-represented by those that have been conducted in cereals (Lin et al., 2012). However, in legumes, “studies of the identification of pod-shattering genes lag far behind those of the cereal crops” (Li and Olsen, 2016).

The shattering system of legume crops is distinct from that of cereals (Li and Olsen, 2016). In legumes, dehiscence is subsequent to the “hygroscopic movement” of the pod valves following dehydration. The release of the accumulated elastic tension during dehydration results in the splitting of the valves along their suture lines (Elbaum and Abraham, 2014). The ability to undergo this movement has often been attributed to specific patterns of lignification of the pod-valve tissues.

Among legumes, The most relevant studies on pod dehiscence have been conducted in soybean. Histological analysis has shown that shattering wild genotypes differ from non-shattering varieties in terms of the degree of lignification of the cells along the suture lines of the pod valves (Dong et al., 2014). Among the cultivated germplasm, differential lignification of the lignin-rich inner sclerenchyma of the pod walls also influences the level of shattering (Funatsuki et al., 2014). Single major genes underlying these histological differences have also been cloned (Dong et al., 2014; Funatsuki et al., 2014). The loss of pod dehiscence has been studied to some extent in lupin, chickpea, pigeonpea, pea yardlong bean, and wild cowpea (Ladizinsky, 1979; Muehlbauer et al., 1998; Boersma et al., 2007, 2009; Weeden, 2007; Abbo et al., 2009; Suanum et al., 2016).

In common bean, there are few such data available. The pioneering studies date to almost a century ago (Lamprecht, 1932; Prakken, 1934). These attempted to index pod-shattering resistance not only based on the occurrence of valve splitting (presence/absence), but also depending on the *mode* of shattering; i.e., based on the degree of torsion (twisting/spiral coiling) of the pod valves after dehiscence (Lamprecht, 1932), and on suggested histological differences between shattering and non-shattering types, mainly in the lignification patterns of the valves tissues (Prakken, 1934). Oligogenic (Lamprecht, 1932) and monogenic (Prakken, 1934) bases for the genetic control of this trait were also proposed. Several decades later, in the pioneer study of Koinange et al. (1996) the pod strings locus (St) was mapped on chromosome 2, and it was proposed to control the differences in shattering between the wax snapbean Midas, an Andean commercial cultivar, and the wild Mesoamerican accession G12873 (Koinange et al., 1996). This locus did not co-segregate with two candidate genes *PvSHP1* and *PvIND*, even if *PvIND* is linked to the St locus (Nanni et al., 2011; Gioia et al., 2012).

The aim of this study was to conduct a comprehensive phenotypic investigation of pod shattering in common bean. With this aim, we also set up a phenotyping pipeline that comprises characterization under field conditions, including the chemical composition, and histological analysis of the pod valves. Following this pipeline, we characterized a population of 267 introgression lines (ILs) that were developed *ad-hoc* to study pod shattering in common bean. In more detail, we pursued the following three goals:

1. To dissect the shattering trait into its “components,” as *level* (percentage of shattering pods per plant) and *mode* (percentage of twisting and non-twisting pods per plant). This will answer the question of how the *level* and the *mode* of shattering predict the resistance to (manual) shattering.
2. To test whether the *occurrence, level*, and *mode* of shattering depend on the chemical composition and histological characteristics of the pod valves. This will help to determine the mechanism of shattering, and it will also allow identification of traits that are useful to surrogate or complement the phenotypic characterization. This might be useful for the development of screening methods that are implementable for high-throughput phenotyping platforms (Fiorani and Schurr, 2013).
3. To test the relationships between pod shattering and other plant traits. This will allow the question to be answered in terms of whether pod shattering itself can be treated as a “syndrome” (i.e., a set of correlated phenotypic characteristics), particularly at the pod level, instead of as a single individual trait.

## Materials and methods

### Plant materials

**Figure 1:**
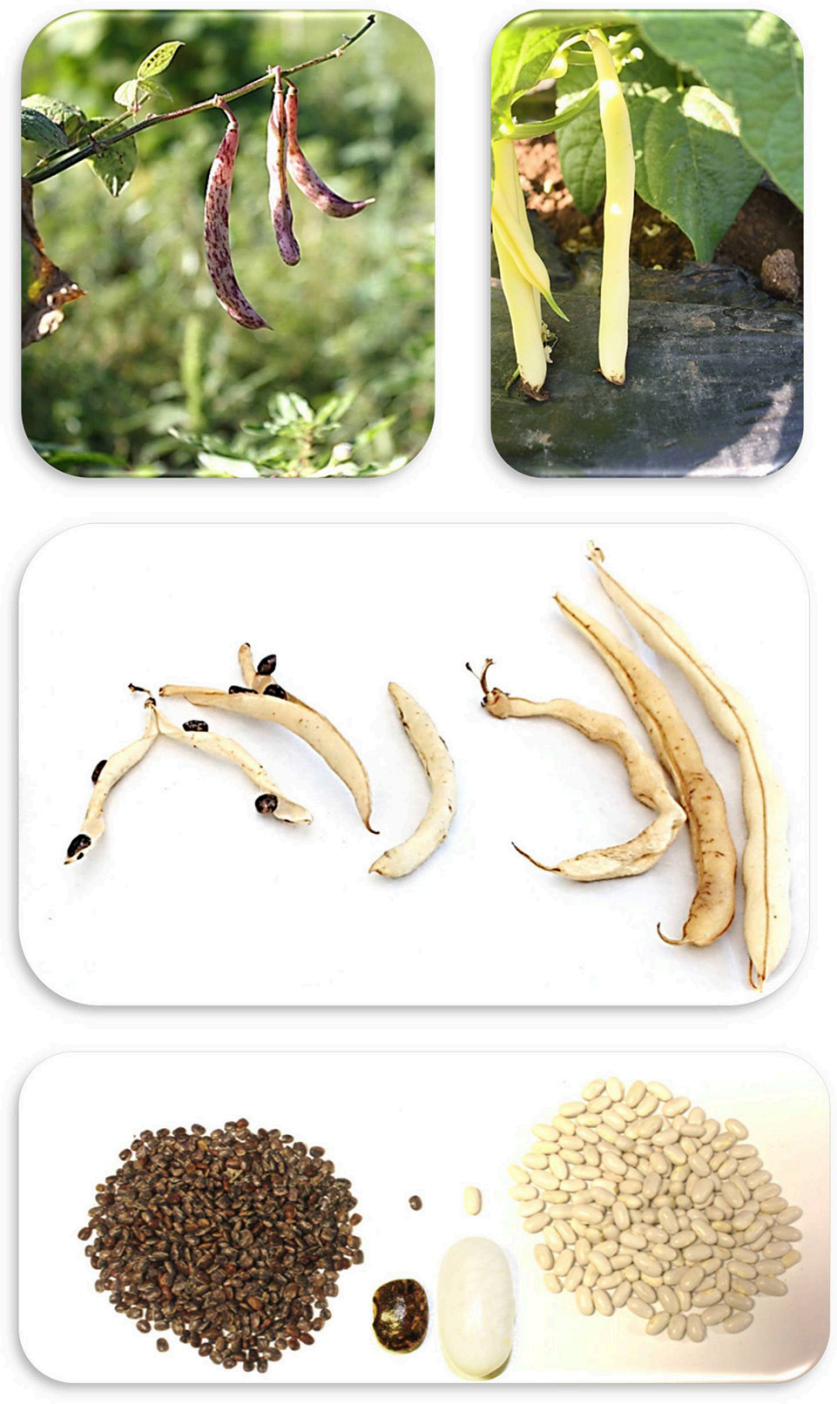
**Representative photographs of the differences between the MG38 (Left)** and MIDAS **(Right)** common beans for pod and seed traits (photographs: M.L. Murgia, D. Rau).

A population of 267 introgression lines was phenotyped, which was representative of a larger set of about 1200 introgression lines developed in the Papa laboratory (Università Politecnica delle Marche, Ancona, Italy) in collaboration with the Attene laboratory (Università degli Studi di Sassari, Sassari, Italy). The population was developed starting from a backcross between the line MG38 belonging to the recombinant inbred line (RIL) population used by Koinange et al. (1996) and the recurrent parent MIDAS (Figure 1). The MG38 line is a RIL obtained from a cross between the wild Mesoamerican genotype, G12873, and the Andean snap bean variety MIDAS. The MG38 genotype was selected for some wild pod traits (small size, curved shape, pigmented valves, pod shattering), and seed characteristics (very small size). However, for other traits (e.g., determinacy, seed dormancy, photoperiod sensitivity), MG38 was selected for domesticated phenotypes to facilitate the population development and increase. Based on amplified fragment-length polymorphism analysis, MG38 has 55% of the genome attributable to the wild Mesoamerica parent G12873 (Papa, unpublished data). MIDAS is characterized by large and relatively straight, yellow and snap bean non-shattering pods, with relatively large seeds.

To obtain the introgression lines, MG38 was back-crossed with MIDAS as a recurrent parent, and different cycles of back-crossing and selfing were carried out together with selection for the wild characteristics of the pods and seeds. Among the 267 lines analyzed in this study, 70 belonged to BC_3_/F_4_:F_5_ families, and 217 to BC_3_/F_6_:F_7_ families. Overall, in the field 130 families were represented. Among these families, 101 families were represented by at least two ILs. In some case, there were three ILs per family (i.e., 29 families were represented by one individual). Precisely, there were 19 BC_3_/F_4_:F_5_ families and 82 BC_3_/F_6_:F_7_ families represented with at least two ILs summing up to 232 ILs.

### Phenotypic characterization

The phenotyping data presented here were obtained in 2014, between May and October (sowing date, May 19). The experimental layout comprised eight rows, each with 35–38 holes; the distance between rows was 1.5 m; the distance between holes (within the rows) was 0.8 m. For each line, a single plant was grown in each single hole. The two parents, MIDAS and MG38, were replicated three times. The positions of the lines were completely randomized. A plastic sheet was used along each row to facilitate weed control (Supplementary Figure 1A). Standard agronomic practices were adopted, in terms of irrigation, fertilization, and pest control. The meteorological conditions were hot and dry with many days with maximum temperature over 30° (Supplementary Figure 1B). Under these conditions, ILs had the opportunity to fully express their shattering phenotype.

#### Measuring pod shattering in the field

**Figure 2:**
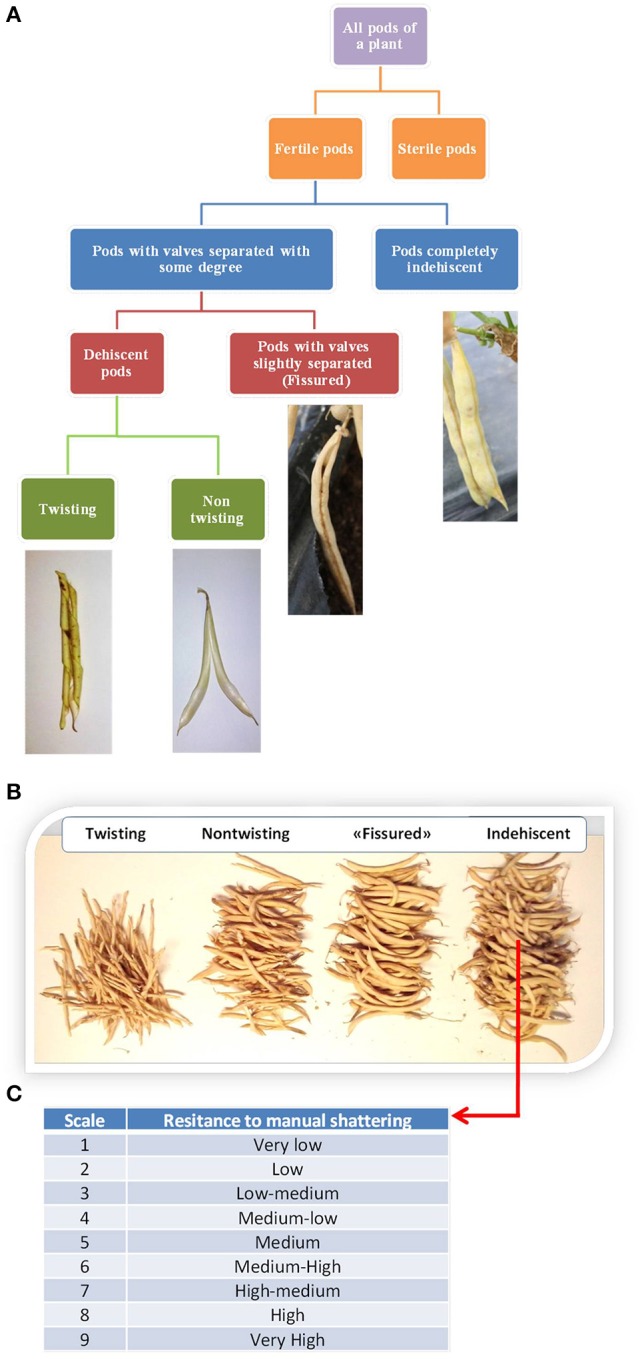
**(A)** Flow diagram of the methods used to classify the pods produced by each plant based on the shattering trait. **(B)** Manual count of the pods produced by the individual plants. **(C)** Classification of the resistance to “manual shattering” of the pods, into discrete scores from 1 (very easy) to 9 (very difficult) (see also Supplementary videos).

We evaluated shattering after each plant reached full maturity. For each plant, we first distinguished between fertile and sterile pods. The numbers of “naturally” shattering and non-shattering pods were then counted. Fertile pods were further classified into different types, as exemplified in Figure 2A. Four pod categories were recognized: Indehiscent; “fissured,” with valves that were not perfectly closed along the ventral suture; dehiscent with non-twisting valves; and dehiscent with twisting valves. It was sometimes difficult to distinguish between these last two categories because of the presence of intermediate cases. Nonetheless, on the basis of this classification, the number of pods falling into each category was counted for each plant (Figure 2B). For the statistical analysis, the number of pods was expressed as the percentage of the fertile pods produced by each plant.

Furthermore, for each line separately, the shattering of indehiscent pods was promoted by hand, for the evaluation of the “resistance to manual shattering,” based on a scale from 1 (i.e., very low resistance to shattering, where valves abruptly shattered under very light pressure on the distal part of the pod) to 9 (i.e., very high resistance to shattering, where valves did not separate and it was necessary to “break” them) (Figure 2C; see also Supplementary Information and videos). To avoid bias, the determination of the resistance to manual shattering was performed independently (i.e., at a different time) from the pod classification.

#### Chemical characterization of the pod valves

The chemical composition of the pod valves was investigated to determine whether the pod shattering was correlated to these characteristics. This element composition analysis looked at carbon, hydrogen, and nitrogen. Here, for each introgression line, 2 g dried pod valves was pulverized in a grinder (18,000 rpm, 1 min). The pulverized tissue was transferred into plastic 50-mL tubes and stored for a few days at room temperature in a cool, dry place. The analyses were performed using 0.080 g pulverized tissue from each line. The samples were combusted at 1,000°C in an excess of oxygen using an element analyser (LECO CHN 628; Leco Corporation, St. Joseph, MI, USA), to determine the carbon, hydrogen, and nitrogen contents. The instrument was calibrated using the “oat meal 502276” forage standard with 46.43% carbon and 2.64% nitrogen. For each run, three independent samples of the standard were included.

The analysis was first performed considering the two parental lines, MIDAS and MG38, with each as three biological replicates (i.e., three plants were grown for each parent). For each biological replicate, there were three technical replicates (i.e., three independent analyses). As there were highly significant differences between the two parents (see Results), the analysis was extended to all of the introgression lines. Three technical replicates were performed for each introgression line.

#### Cell-wall analysis

For each individual plant, 6 g dried valves were pulversed in a mill (Retsch SM 100) for 10 min. The procedure of Van Soest and Wine (1967) was then followed. First, the neutral detergent fiber was quantified, which represents the total content in the cell wall of the analyzed sample. Thus, the acid detergent fiber was determined, which mainly represents an intermediate step that is necessary to extract the acid detergent lignin, which correlates with the lignin content of the sample analyzed. We also calculated the differences for the neutral detergent fiber minus the acid detergent fiber, and the acid detergent fiber minus the acid detergent lignin, which provided rough estimations of the hemicellulose and cellulose contents, respectively (Van Soest and Wine, 1967). All of these chemical fractions are expressed as percentages of the dried organic matter after subtracting the weight of the ashes (see Supplementary Information for further details).

This analysis was initially performed for MIDAS and MG38, for which three biological replicates were available. For each biological replicate, three technical replicates were included. As the analysis of variance (ANOVA) showed clear-cut differences between the parent lines, the cell-wall analysis was extended to 12 indehiscent introgression lines, and 12 high-shattering introgression lines (>65% shattering, as seen for MG38, the wild-like parent).

#### Anatomical and histological study of the pod valves

This study had the specific aim to look for differential patterns between the shattering and non-shattering lines, particularly for lignin deposition. The analysis was conducted considering 5- and 20-days-old pods, and pods at the maturation stage. The pods were kept in a solution of 95% ethanol and glacial acetic acid (5:2, v/v) for 3 days, and then stored at 4°C in 70% ethanol. Sections of the ventral and dorsal suture sheath were obtained manually. The sections were treated with Javelle water (an aqueous solution containing sodium hypochlorite and some sodium chloride, used as a bleach and disinfectant) for ~10 min. After this washing, the sections were immersed in 50% acetic acid for a few minutes.

The pod valves were also embedded in paraffin, and 10-μm sections were obtained using a sliding microtome (Reicher) (see Supplementary Information for further details). The manually obtained sections were stained according to two different methods: Toluidine blue O (TBO), and carmine-iodine green; whole microtome sections were stained only with toluidine blue O. The toluidine blue O was used to differentially stain polysaccharides and lignin, whereby cells with thick lignified walls are sky blue, and cellulose and hemicellulose are dark blue (Mitra and Loqué, 2014). With carmine-iodine, lignin is green, and cellulose is pink (Deysson, 1954).

#### Phenotyping of the other plant characteristics

To allow the study of the relationships between shattering and the other plant traits, a total of 27 traits were recorded (7 qualitative, 20 quantitative). These were: Number of cotyledonary leaves (two, three); angle of the cotyledonary leaves (60°, 120°, 180°); lobature of the cotyledonary leaves; stem color (green, red); growth type (non-climbing, intermediate, climbing); flower color (white, light purple, dark purple); pod color (yellow, striped, with from 1 to 3 stripes); plant height (cm); plant vigor (height per width; cm^2^); flowering time and pod setting (days from May 19); pod weight per plant (g); valve weight per plant (g); seed weight per plant (g); number of pods per plant; number of seeds per plant; mean pod weight (g); mean valve weight (g); 100-seed weight (g); weight of seeds per pod (g); number of seeds per pod; Harvest Index at pod level. To avoid loss of seeds at the maturation stage in the shattering plants, mature pods were (almost) enveloped in plastic nets (Supplementary Figure 2). Moreover, at the end of the ripening stage (i.e., before shattering occurred), 10 pods per introgression line were randomly sampled. These were scanned, and the acquired images (600 dpi) were processed with the Tomato Analyzer software (Rodríguez et al., 2010), to determine the following pod traits: Perimeters; area; curved height; maximum height; maximum width (Supplementary Figure 3). All of these measures were in pixels. We also calculated the ratio of the curved length to maximum height, where a ratio of 1.0 indicates a perfectly straight pod, while ratios <1 indicate a more or less marked “C” shapes of the pods. All of these variables must be referred to the projection area of the pod on the scanner glass. The procedure was first set up for the parental lines, MG38 and MIDAS. The analysis was then extended to all of the other introgression lines. For statistical analysis, 10 pods per introgression line were considered, and the means were calculated.

### Statistical analysis

For each variable used to describe shattering, the frequency distribution was first determined. Associations between variables were quantified using Pearson “*r*” coefficient (quantitative traits) or contingency analysis (qualitative traits). Differences among groups of lines for the various phenotypic and chemical traits were tested using one-way analysis of variance (ANOVA), considering each line as a “replicate” of the group.

Resistance to manual shattering was modeled based on the other six indicators of pod shattering: Indehiscent (%); valves separated to some degree (%); fissured (%); shattering (%); non-twisting (%); twisting (%) (see Figure 2). With this aim, the method of recursive partitioning was adopted, which is also known as decision-trees analysis. This is particularly indicated to investigate relationships among variables without having an *a*-*priori* model, and it is particular powerful as it considers a very high number of possible partitions, and takes into consideration only the best one (see JMP version 7, User Manual; SAS Institute Inc., Cary, NC, USA). In this case, the categorical X variable was the degree of “resistance to manual shattering” (scored as 1 to 9), while all of the other six indicators of shattering were considered as possible explanatory Y variables. Thus, it was possible to obtain a hierarchal system of (dichotomic) criteria that allowed the prediction of the manual shattering *resistance* from the observed *level* and *mode* of shattering. Statistical analysis of the phenotypic data was all performed using JMP version 7 (SAS Institute Inc., Cary, NC, USA).

## Results

### Shattering *level* and *mode*

As expected, the two parental lines showed highly contrasting phenotypes for pod shattering: MIDAS was completely indehiscent, while MG38 was highly dehiscent, with a mean of 65% shattering pods per plant. Moreover, 98% of the variance for shattering occurrence was located among-families indicating a very limited role of environmental factors influencing this trait in the population of ILs grown under our field conditions (see Supplementary Information and Supplementary Table 1).

Table 1 gives the descriptive statistics for the six variables measured to characterize the introgression lines for the pod-shattering trait.

**Table 1:** **Descriptive statistics for the six variables used to measure the pod shattering trait in the population of bean introgression lines**.

**Shattering trait**	**Statistics**			
	**Mean (%)**	**Standard deviation (%)**	**Standard error of the mean**	**95% confidence interval**
Completely non-dehiscent	50.4	26.7	1.6	47.2–53.6
Valves separated to some degree	49.6	26.7	1.6	46.4–52.8
– Fissured	18.0	13.4	0.8	16.4–19.7
– Dehiscent	31.6	21.3	1.3	29.1–34.1
– Twisting	11.5	10.9	0.7	10.2–12.8
– Non-twisting	20.1	14.3	0.9	18.4–21.8

The introgression lines were highly variable for the pod-shattering trait, as the indehiscent pods per plant ranged from 3.9 to 100%, with a mean of 50.4%. Twenty-nine introgression lines (~10% of the total) were completely indehiscent. The pods per plant with valves separated to some degree ranged from 0 to 96.1%, with a mean of 49.6%. The distribution of these two variables tended to bimodality (Supplementary Figure 4). The fissured pods per plant ranged from 0 to 71.7%, with a mean of 18.0%.

The *levels* of shattering were highly variable, as the shattering pods per plant ranged from 0 to 82.6%, with a mean of 31.6%. The *modes* of shattering were also highly variable, as the non-twisting and twisting pods per plant both ranged from 0 to ~60%. Non-twisting pods were more frequent than twisting pods, with means of 11.1 and 20.1%, respectively.

**Figure 3:**
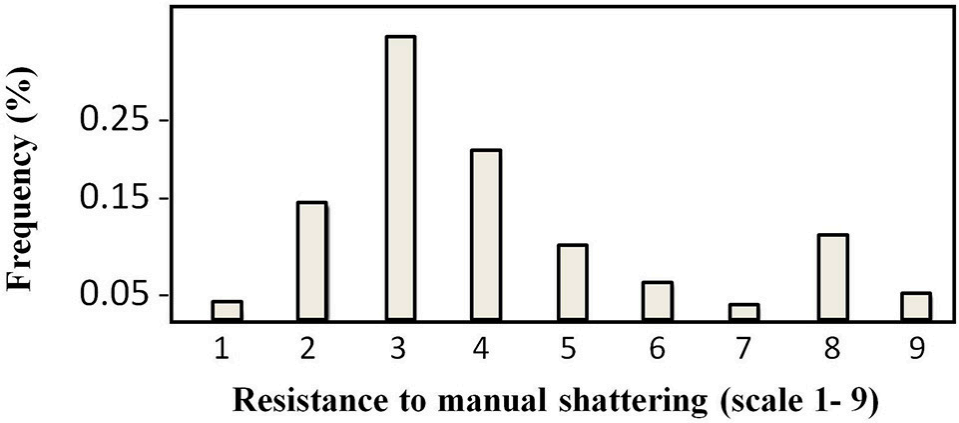
**Frequency distribution of the scores for resistance to manual shattering**. Scores are from 1 (very easy) to 9 (very difficult) (see Figure 2C).

The distribution of the trait “resistance to manual shattering” is illustrated in Figure 3. MIDAS had a score of 8 (i.e., high resistance), while MG38 had a score of 2 (i.e., low resistance). The mean for this trait was 4.12 (i.e., medium-low resistance; σ = 1.96; S.E. = 0.12), and the distribution appeared to be bimodal. About 15% of the introgression lines showed scores of 1 and 2 (i.e., ≤ MG38), while about 10% showed scores of 8 and 9 (i.e., ≥MIDAS).

#### Relationships among the measures of shattering

**Figure 4:**
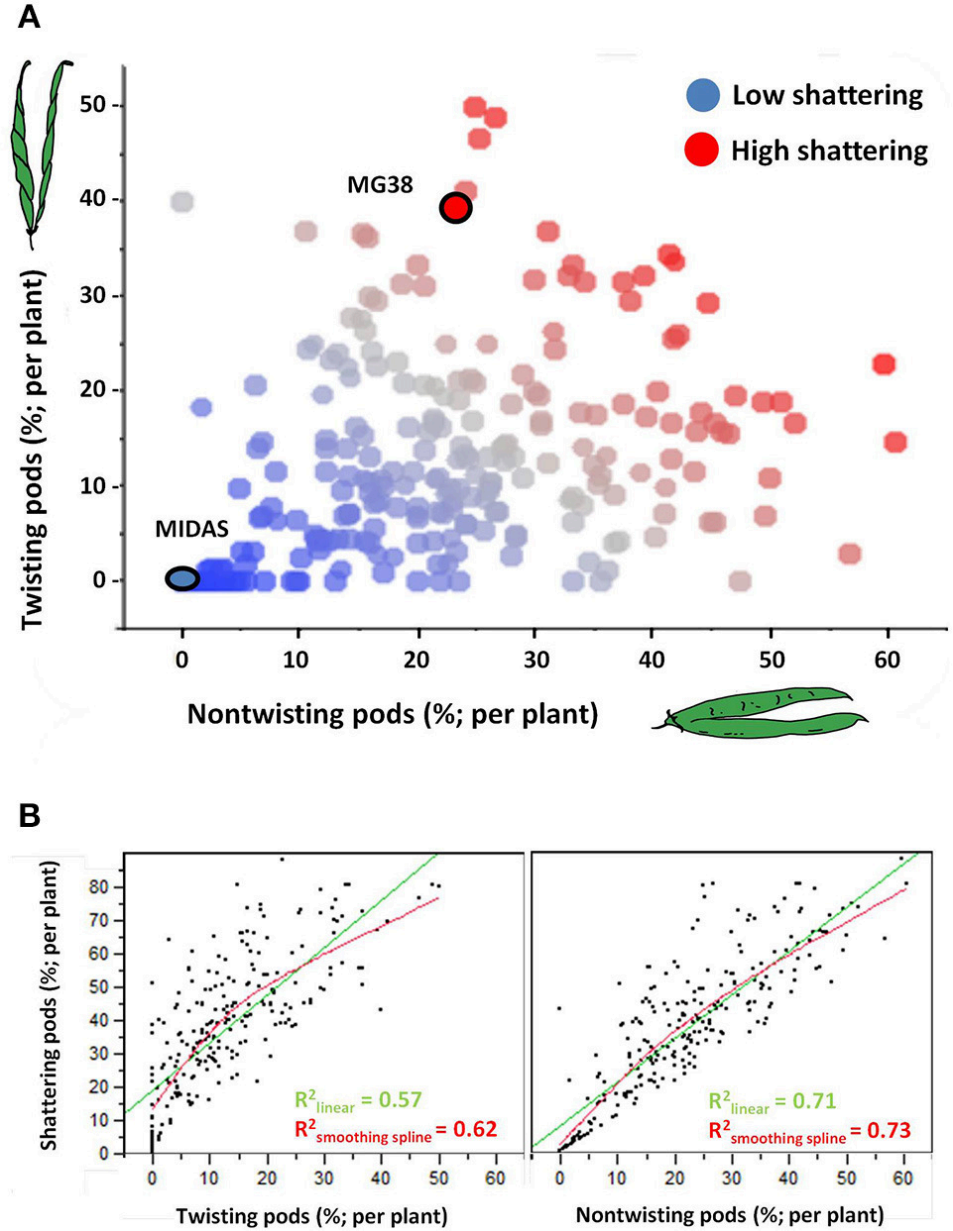
**(A)** Relationship between level of shattering (percentage shattering pods per plant) and mode of shattering (percentages twisting and non-twisting). Deep blue, no shattering; red, high shattering. **(B)** Left: Association between levels of shattering and frequencies of twisting pods. Right: Association between levels of shattering and frequencies of non-twisting pods. In both cases, linear and smoothing spline (λ = 10,000) fits are shown. The associations were tested excluding the completely indehiscent plants.

Figure 4A shows the relationships between the *levels* and *modes* of shattering. Introgression lines with the same or very similar levels of shattering (percentage shattering pods per plant) showed a very different ratio between the twisting and non-twisting types. For example, among the introgression lines with very high levels of shattering (>65%), the ratio of non-twisting to twisting pods per plant varied from ~25:50 (1:2) to about 60:15 (4:1). Moreover, these data also suggested that transgressive variation probably occurred for ~10% of the lines, which showed higher shattering than MG38 (>65%), the highly shattering parental line (Figure 4A).

The level of shattering was more strongly correlated with the frequency of non-twisting pods (*R*^2^ = 0.71, *P* < 10^−4^) than with the frequency of twisting pods (*R*^2^ = 0.57; *P* < 10^−4^) (Figure 4B). In particular, while a low number of twisting pods corresponded to different levels of shattering, a low number of non-twisting pods was more indicative of low levels of shattering.

**Figure 5:**
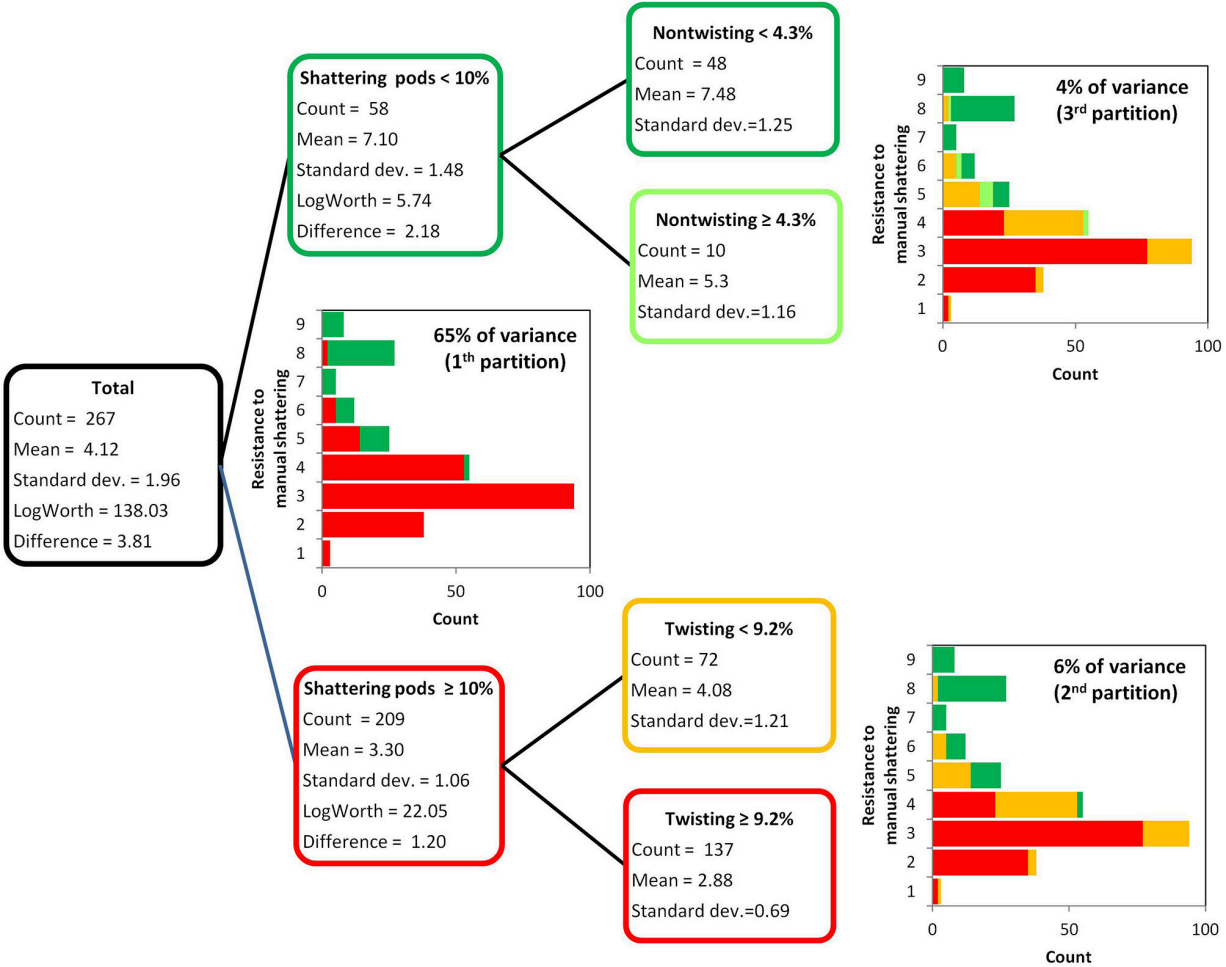
**Results of the recursive partition analysis**. Resistance scores for manual shattering varied from 1 (low resistance) to 9 (high resistance). The first partition identified two groups of introgression lines (with <10% and with ≥10% shattering pods). Within each of these two groups, the second and third partitions identified two further subgroups, based on the percentages of twisting and non-twisting pods, respectively.

The resistance to manual shattering was modeled considering the six variables measured to dissect out the shattering trait (Figure 2, Table 1), with recursive partition analysis applied (Figure 5). The variable that best predicted resistance to manual shattering was the shattering pods per plant; i.e., the *level* of shattering. Indeed, the threshold of 10% shattering pods defined two groups of plants with different mean shattering resistance scores of 3.3 and 7.1; this partition captured 65% of the total variance for shattering resistance (*P* < 0.0001; Figure 5). A second partition suggested a role for the *mode* of shattering. Indeed, within the group of introgression lines showing < 10% shattering pods per plant, a threshold of 9% twisting pods defined two subgroups of plants that had mean shattering resistance scores of 2.9 and 4.1. This partition captured a small portion, 6%, of the total variance for resistance to manual shattering (*P* < 0.0001; Figure 5). A third partition was found within the group of introgression lines with shattering pods ≥10%. Here, the threshold of 4.2% of non-sigaroid pods defined two subgroups of plants with mean shattering resistance scores of 5.3 and 7.5, and these explained an additional 4% of the total variance for shattering resistance (Figure 5). Thus, cumulatively these three partitions explained 75% of the total variance. The fourth partition (not shown) explained 0.8% of the total variance for shattering resistance, which indicated that dealing with a more complex model was not necessary.

### Chemical analysis

The pod valves of the two parental lines, MG38 and MIDAS, had significantly different carbon contents (ANOVA, *P* < 0.0001; Table 2). The highly dehiscent MG38 had a carbon content of 43.8% dry weight, which gave a 6.8% increase in the carbon content of the indehiscent MIDAS, from 41.0% dry weight (Table 3). ANOVA also revealed a marginally significant difference for the hydrogen contents (*P* < 0.047; Table 2), again in favor of MG38 (6.7% dry weight) compared to MIDAS (6.5% dry weight; Table 3). The difference in the nitrogen contents was not significant (*P* = 0.502) (Tables 2, 3).

**Table 2:** **Results for the ANOVA performed for the chemical element analysis of the pod valves**.

**Element**	**MIDAS vs. MG38**				**Indehiscent vs. dehiscent**				<**7.14 vs**. ≥**7.14% shattering**			
	**M.S**.	***F*****_1, 4_**	***P***	**$R_{adj}^{2}$**	**M.S**.	***F*****_1, 227_**	***P***	**$R_{adj}^{2}$**	**M.S**.	***F*****_1, 227_**	***P***	**$R_{adj}^{2}$**
Carbon	12.30	256.0	< 0.0001	0.92	191.93	105.2	< 0.0001	0.31	286.04	202.92	< 0.0001	0.47
Hydrogen	0.04	8.1	0.047	0.15	0.72	6.7	0.010	0.02	0.29	2.63	0.110	0.01
Nitrogen	0.00	0.5	0.502	0.00	0.10	2.3	0.130	0.01	0.05	1.34	0.250	0.00

*ANOVA was applied to the following comparisons: Parental lines MG38 (dehiscent) vs. MIDAS (indehiscent); indehiscent vs. dehiscent introgression lines; and introgression lines with <7.14 vs. ≥7.14% shattering pods. M.S., Mean Square; F_x/y_, F ratio with x and y degrees of freedom for the numerator and denminator, respectively. $R_{adj}^{2}$, adjusted R^2^; P, significance level*.

**Table 3:** **Mean contents of carbon, hydrogen, and nitrogen of the pod valves as applied to the following comparisons: two parental lines MG38 (dehiscent) vs. MIDAS (indehiscent); indehiscent vs. dehiscent introgression lines; and introgression lines with < 7.14 vs. ≥7.14% shattering pods**.

**Element**	**Comparison**	***N***	**Statistic (% dry weight)**		**Comparison**	***N***	**Statistic (% dry weight)**		**Comparison**	***N***	**Statistic (% dry weight)**	
			**Mean**	**95% confidence interval**			**Mean**	**95% confidence interval**			**Mean**	**95% confidence interval**
Carbon	Midas	3	41.0	40.7-41.4	Indehiscent	26	41.7A	41.2–42.2	< 7.14%	41A	41.8	41.5–42.2
	MG38	3	43.9	43.5-44.2	Dehiscent	203	44.6B	44.4–44.8	≥7.14%	188B	44.8	44.6–44.9
Hydrogen	Midas	3	6.5	6.4-6.6	Indehiscent	26	6.3A	6.2–6.5	< 7.14%	41	6.4	6.3–6.5
	MG38	3	6.7	6.6-6.8	Dehiscent	203	6.5B	6.5–6.6	≥7.14%	188	6.5	6.5–6.6
Nitrogen	Midas	3	0.6	0.5-0.8	Indehiscent	26	0.5	0.5–0.6	< 7.14%	41	0.5	0.5–0.6
	MG38	3	0.6	0.5-0.7	Dehiscent	203	0.5	0.5–0.5	≥7.14%	188	0.5	0.5–0.5

*Groups of introgression lines with different letters have significantly different means (P < 0.05; Tukey-Kramer multiple comparison tests)*.

The comparison of the indehiscent vs. dehiscent introgression lines was highly significant for the carbon contents (Table 2). The dehiscent introgression lines showed a 6.9% increase in the carbon content of the indehiscent introgression lines, according to dry weight (Table 3). The indehiscent introgression lines had the same carbon content as MIDAS, while the dehiscent introgression lines had the same carbon content as MG38. The difference between these dehiscent and indehiscent introgression lines was small, but significant for the hydrogen content although not for the nitrogen content (Tables 2, 3). The frequency distribution for the carbon contents tended to be bimodal, while this was less evident for the hydrogen and nitrogen contents (Supplementary Figure 5).

**Figure 6:**
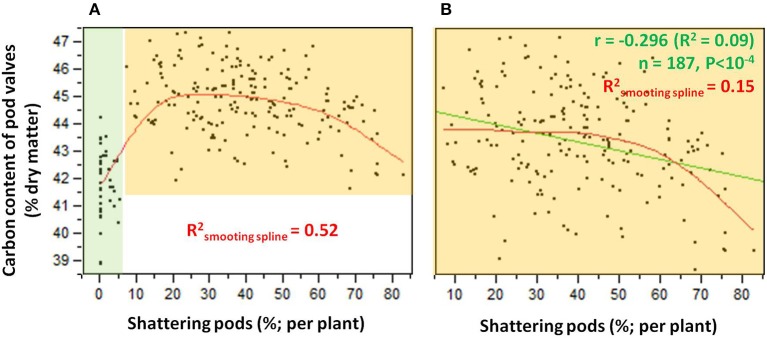
**(A)** Relationship between carbon contents and levels of shattering. Green shading, individuals for which shattering was <7.14%; orange shading, individuals for which shattering was ≥7.14%. **(B)** Relationship between carbon contents and shattering levels excluding individuals with low or no shattering. *R*^2^ given for smoothing spline (λ = 10,000) (red) and linear fit (green).

The relationship between the carbon contents and the shattering pods per plant (Figure 6) showed an abrupt transition in the carbon content that occurred between 5 and 10% shattering pods per plant (Figure 6A). Partition analysis showed that this transition occurred at 7.14% shattering pods per plant (not shown). The definition of the introgression lines into two groups based on this transition, with the first with < 7.14% and the second with ≥7.14% shattering pods per plant, captured 47% of the total variance for the carbon contents (Table 2). When the introgression lines with < 7.14% shattering pods were excluded from the analysis, there was a weak, but significant, negative correlation (*r* = −0.296; *P* < 0.0001) between shattering level and carbon content (Figure 6B).

### Cell-wall analysis

**Figure 7:**
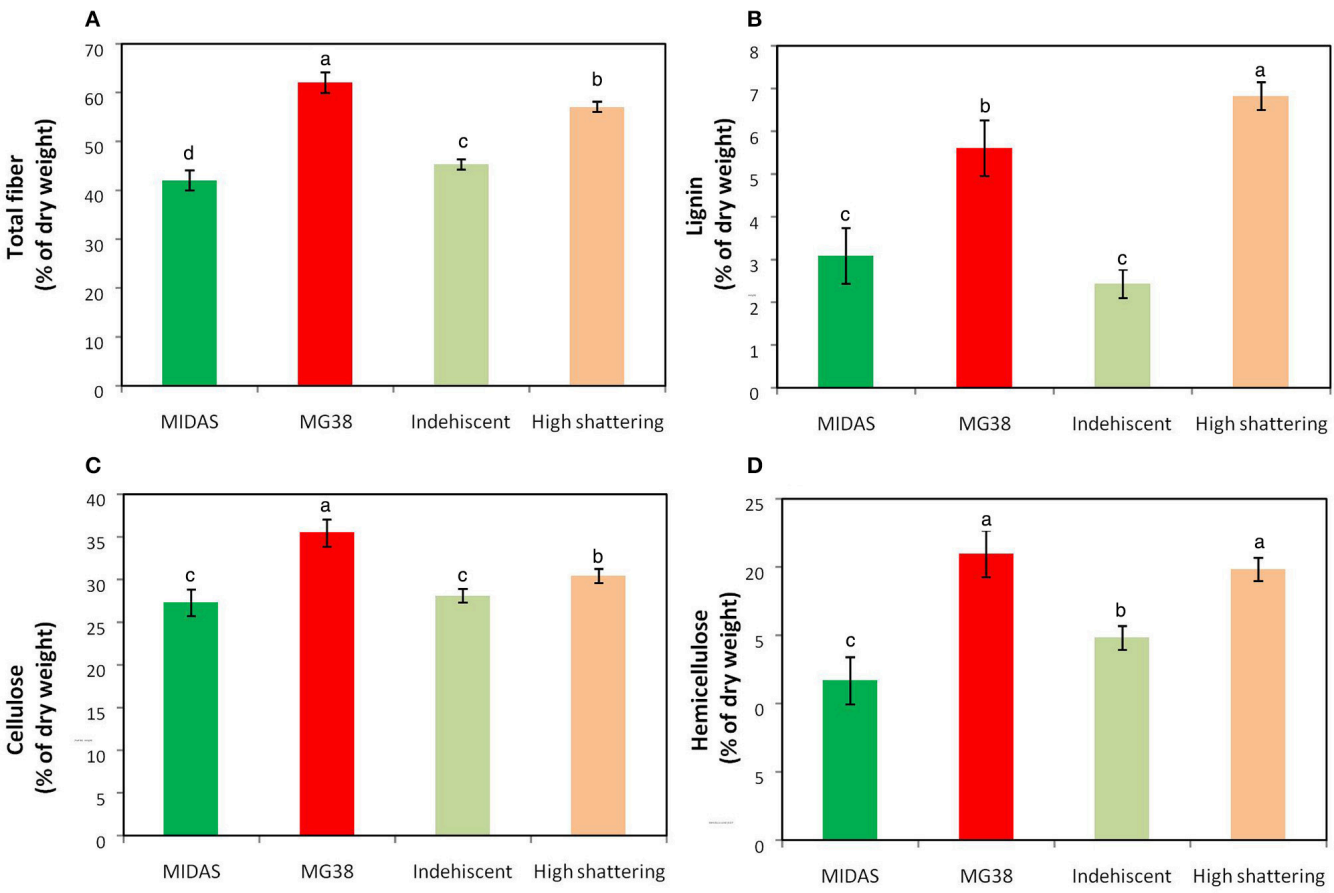
**Results of cell-wall analysis**. **(A)** Total fiber, **(B)** Lignin, **(C)** Cellulose and **(D)** Hemicellulose composition. Comparisons between parental lines MIDAS (indehiscent) and MG38 (highly dehiscent), and between nonshattering and highly shattering introgression lines. Each group of introgression lines comprised 12 individuals. The group of highly shattering introgression lines had percentages of shattering pods >MG38 (>65%). Green, MIDAS; red, MG38; light green, non-shattering introgression lines; light red, highly shattering introgression lines. Columns with different letters have significantly different means *(P* < 0.05; Tukey-Kramer multiple comparison tests).

The pod valves of MIDAS and MG38 had significantly different total fiber contents (ANOVA, *P* < 0.0001; Table 4). The fiber content of the highly dehiscent MG38 (62.0%) showed a ~48% increase of that of the completely indehiscent MIDAS (42.0%; Figure 7A). The contents of the three cell-wall components of lignin, hemicellulose, and cellulose were always higher for MG38 than MIDAS, with the greatest difference seen for lignin, followed by hemicellulose and cellulose (Figures 7B–D). Statistical analysis was carried out to compare the two groups of introgression lines, the first comprising the completely indehiscent plants (i.e., non-shattering), and the second including the plants with >65% shattering pods per plant (i.e., high shattering) (Table 4, Figures 7A–D). These two groups strongly differed in their lignin contents, with the difference for the latter representing a 180% increase of the former, which was much greater than the increase from the MIDAS to MG38 parental lines (80%) (Figure 7B). The high shattering group also showed increases of the non-shattering group for hemicellulose (33.9%) and cellulose (7.6%) contents, which were here less than for the parental lines (79.5%, 30.1%, respectively) (Figures 7C,D). These highly dehiscent (i.e., high shattering) introgression lines showed lower total fiber content than MG38 (Figure 7A), which was mainly due to reduction in the cellulose content, and to a slight, although not statistically significant, reduction in the hemicellulose content (Figure 7D). In contrast, these lines had higher lignin content compared to MG38 (Figure 7B). This suggested that the achievement of the very high pod shattering ability (here even higher than the wild-like MG38) is associated with an increase in the proportion of lignin in the cell wall.

**Table 4:** **Results for the ANOVA performed for the cell-wall analysis**.

**Cell-wall component**	**Midas vs. MG38**				**Indehiscent vs. highly dehiscent**			
	**M.S.**	***F*****_1, 4_**	***P***	**$R_{adj}^{2}$**	**M.S**.	***F*****_1, 22_**	***P***	**$R_{adj}^{2}$**
Total fiber (NDF)	600.2	98.7	<10^−3^	0.95	825.15	273.04	<.0001	0.92
Lignin (ADL)	9.5	163.0	<10^−3^	0.97	115.74	305.35	<.0001	0.93
Hemicellulose (NDF-ADF)	129.3	14.0	0.02	0.72	151.43	140.95	<.0001	0.86
Cellulose (ADF-ADL)	100.9	134.1	<10^−3^	0.96	32.05	14.64	0.0009	0.37

*ANOVA was applied to the following comparisons: Parental lines MG38 (dehiscent) vs. MIDAS (indehiscent); and two groups of 12 introgression lines, one completely indehiscent vs. one highly dehiscent (i.e., with percentage shattering pods >65% of MG38). M.S., Mean Square; F_x/y_, F ratio with x and y degrees of freedom for the numerator and denominator, respectively. $R_{adj}^{\text{2}}$, adjusted R^2^; P, significance level*.

*NDF, neutral detergent fiber; ADL, acid detergent lignin; ADF, acid detergent fiber*.

### Correlations between the element compositions and the cell-wall analysis

The carbon content was strongly correlated with the total fiber content of the pod valves (*r* = 0.685); moreover, among the three cell-wall components (i.e., lignin, hemicellulose, cellulose), the carbon content showed the best correlation with the lignin content (*r* = 0.672; Table 5). Stepwise multiple regression analysis was performed with the carbon content as the dependent variable and lignin, hemicellulose, and cellulose as the independent variables (Supplementary Table 2). Here, the only variable that entered into the model was the lignin content (*P* = 1.85 × 10^−5^). This thus indicates that the hemicellulose and cellulose correlation to the carbon content was mainly due to their correlation with the lignin content.

**Table 5:** **Correlations between the element compositions and cell-wall fiber contents for the pod valves of the 24 introgression lines, 12 non-shattering, and 12 very high shattering**.

**Cell-wall component**	**Element composition**		
	**%Carbon**	**%Hydrogen**	**%Nitrogen**
Total fiber (NDF)	0.685^***^	0.038	0.218
Lignin (ADL)	0.672^***^	0.081	0.247
Hemicellulose (NDF-ADF)	0.623^**^	0.140	0.195
Cellulose (ADF-ADL)	0.527^*^	−0.183	0.134

NDF, neutral detergent fiber; ADL, acid detergent lignin; ADF, acid detergent fiber;

* P < 0.05;

** P < 0.01;

*** *P < 0.001*.

### Anatomical and histological analysis of the pod valves

The analysis conducted with 5-day-old pods showed no obvious differences between the shattering and non-shattering genotypes (Supplementary Figure 6). The ventral sheath showed only a few cells with very low levels of lignification. A similar situation was observed for the dorsal sheath (not shown). There was no lignin deposition in correspondence with the inner parenchyma cells of the pod walls.

**Figure 8:**
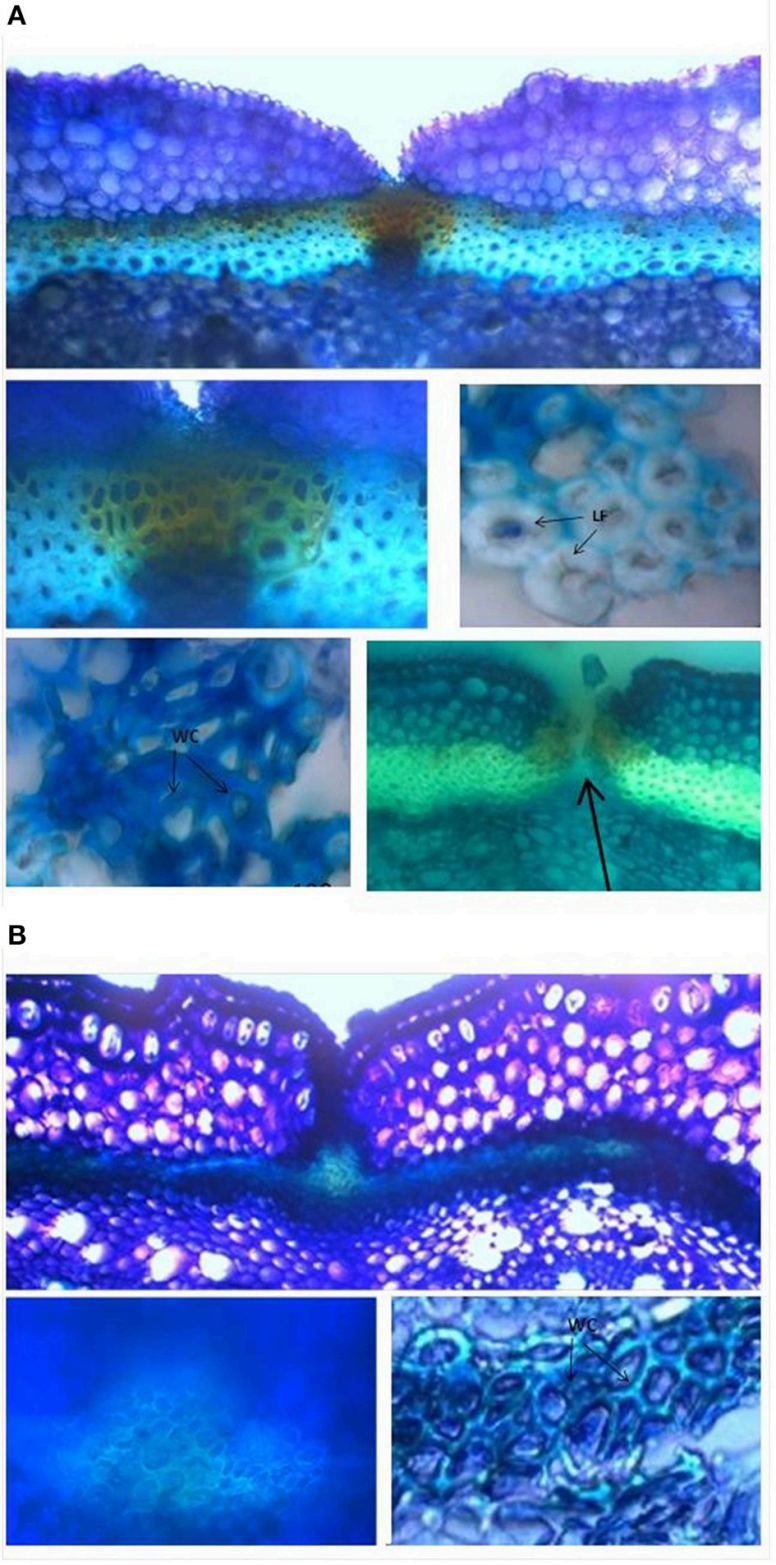
**Representative images from the histological study of the ventral sheath of the pod valves**. **(A)** toluidine blue O (TBO) staining, showing ventral sheath of pod valves from MG38 (highly dehiscent), and details of dehiscent zone, thick lignified fibers (sclerenchyma), wood cells, dehiscent zone after cracking (arrow). **(B)** TBO staining, showing ventral sheath of pod valves from MIDAS (indehiscent), with two details of the indehiscence zone. LF, lignified fibers; WC, wood cells.

**Figure 9:**
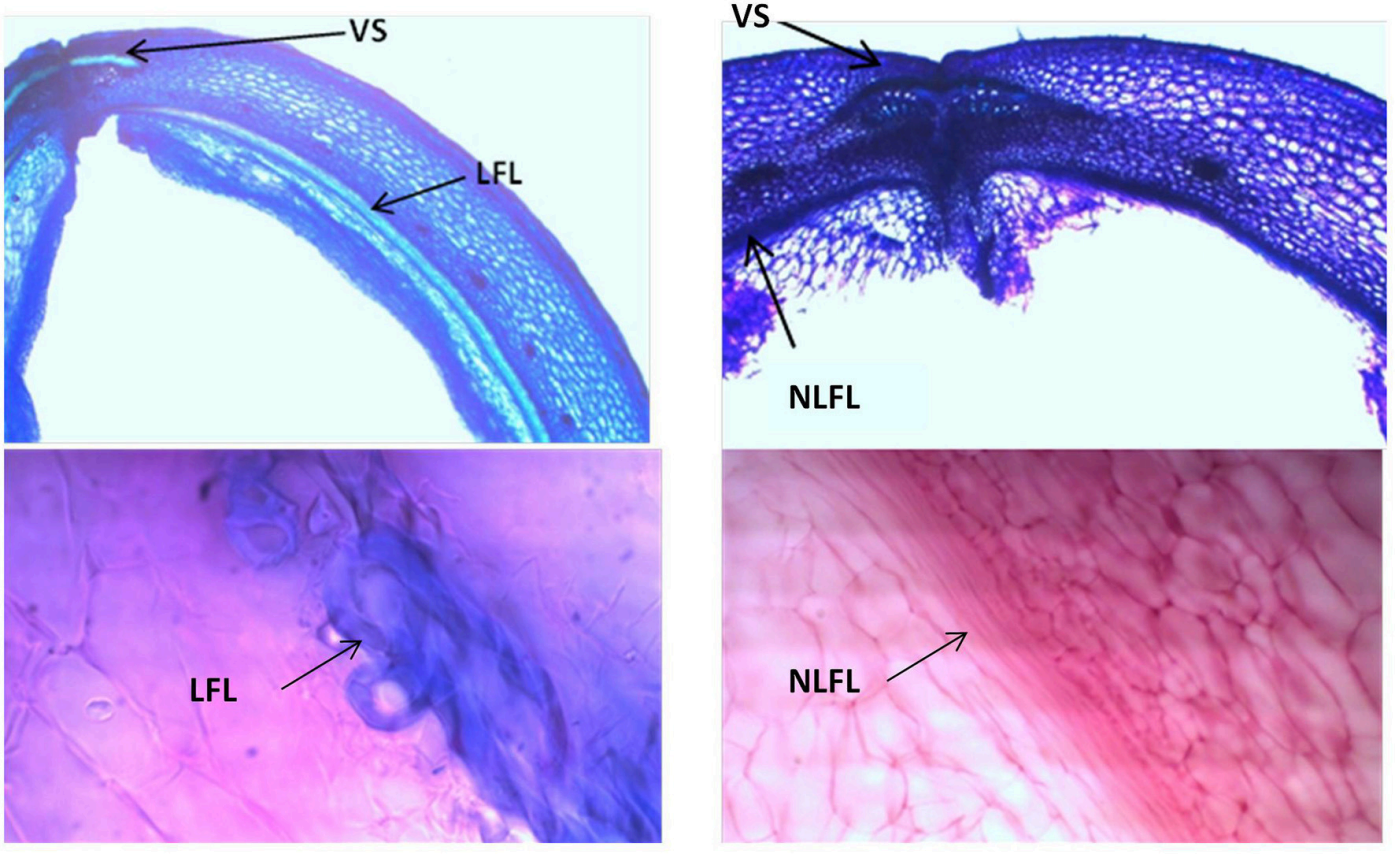
**Representative images with toluidine blue O (TBO) (Upper panels)** and carmine-iodine green staining **(Lower panels)**, illustrating differences between pod valves from MG38 (shattering; left) and MIDAS (non-shattering; right) in terms of the degree of lignification of the inner layer of the sclerenchymatic cells of the pod wall. VS, ventral sheath; LFL, lignified fiber layer; NLFL, non-lignified fiber layer.

Analyses of 20-day-old pods showed evident lignin deposition in the ventral sheath of the pod valves, and a clear-cut difference between the shattering (i.e., MG38) and non-shattering (i.e., MIDAS) genotypes (Figures 8A,B). Indeed, the proportion of cells with thick secondary cell-wall formation (i.e., sclerenchymatic cells) was clearly greater for MG38 (highly dehiscent), compared to MIDAS (indehiscent). The absence of cells with thick secondary cell-wall formation for MG38 was limited only to the external layer of the cells of the sheath and to the dehiscence zone, while for MIDAS this involved all of the sheath (Figure 8B). Moreover, for MG38, the cell-wall thickness tended to reduce when moving from the sheath to the dehiscence zone (Figure 8A), where there was the tendency to easily “fracture” (Figure 8A). A similar pattern was observed for the dorsal sheath (Supplementary Figure 7). A clear-cut difference was also seen between the parental MG38 and MIDAS for the degree of lignification in the inner cells of the pod walls, with very strong lignification (i.e., sclerenchyma) for MG38, and complete absence of lignin deposition for MIDAS (Figure 9).

As not all anatomical or histological differences between the wild-like parent (MG38) and the cultivated varieties (MIDAS) are necessarily correlated with the shattering traits, two introgression lines (one with shattering >MG38, the other without shattering) were also compared (Supplementary Figures 8A–C). Encouragingly, here the patterns were similar to those observed between MG38 and MIDAS, which suggests that the histological differences seen do indeed underlie the shattering/non-shattering phenotypes.

It was difficult to obtain good sections of the pod valves at the maturation stage because of the fragility of the tissue. However, it can be noted that at this stage, the ventral sheath of MIDAS had more mechanical resistance than that of MG38, which appeared to be very fragile instead (Supplementary Figure 9).

### Relationships between pod shattering and the other plant characteristics

Table 6 gives the associations between the levels of shattering (percentage shattering pods per plant) and the other 28 phenotypic traits, of which seven are qualitative and 21 are quantitative. These included morphological, phenological, and productive traits. Overall, shattering was very poorly correlated with all of these plant traits considered. Significant weak associations were detected for three qualitative traits, four quantitative–productive traits, and six precision phenotyping traits that describe pod size and shape (Table 6).

**Table 6:** **Correlations between level of shattering (percentage shattering pods per plant) and the 28 phenotypic traits**.

**Trait**	**Association**			
**Qualitative**	**$R_{adj}^{2}$**	**M.S**.	***F***	***P***
Number of cotyledonary leaves	0.000	106.86	0.24	0.628
Angle of cotyledonary leaves	0.000	99.21	0.21	0.810
Lobature of cotyledonary leaves	0.010	672.75	1.48	0.229
Stem color	**0.020**	**2446.21**	**5.50**	**0.020**
Growth type	0.000	15.59	0.04	0.950
Flower color	**0.140**	**8396.23**	**21.45**	<**10**^−4^
Pod color	**0.120**	**4708.32**	**11.75**	<**10**^−4^
**Quantitative**	***R***^2^	***R***	***N***	***P***
Plant height	0.010	0.102	264	0.099
Flowering time	0.000	0.000	266	0.998
Pod setting	0.001	0.034	264	0.058
Pods weight per plant	0.003	0.056	267	0.366
Valve weight per plant	**0.029**	**0.169**	**264**	**0.006**
Seed weight per plant	0.000	−0.003	265	0.949
Number of pods per plant	0.014	0.117	265	0.055
Number of seeds per plant	0.010	0.100	266	0.103
Mean pod weight	0.001	−0.031	267	0.611
Mean valve weight	**0.071**	**0.267**	**264**	<**10**^−4^
100-seed weight	**0.045**	−**0.212**	**265**	**0.001**
Seeds per pod	0.014	−0.119	265	0.052
Number of seeds per pod	0.006	0.770	266	0.211
Harvest Index at the pod level	**0.106**	−**0.327**	**265**	<**10**^−4^
Pod perimeter	**0.089**	−**0.298**	**259**	<**10**^−4^
Pod area	**0.022**	−**0.148**	**259**	**0.017**
Pod maximum width	**0.181**	−**0.425**	**259**	<**10**^−4^
Pod maximum height	**0.020**	−**0.143**	**259**	**0.021**
Pod curved height	**0.071**	−**0.266**	**259**	<**10**^−4^
Pod maximum/ curved height	**0.120**	−**0.347**	**259**	<**10**^−4^

*Bold, significant associations. M.S., Mean Square; F, F ratio; $R_{adj}^{2}$, adjusted R^2^; r, Pearson correlation coefficient; P, significance level*.

For the associations with the qualitative traits, it was observed that: Plants with red stems showed higher shattering (59.6 ± 12.66%) than those with green stems (31.25 ± 1.30%); plants with white flowers showed higher shattering (36.52 ± 1.44%) than those with purple (22.85 ± 2.67%) and light purple (13.66 ± 3.96%) flowers; plants with yellow pods showed higher shattering (35.89 ± 1.44%) than those with striped/yellow pods (17.58 ± 2.80%), with an intermediate position seen for those with striped pods (30.55 ± 6.33%) and yellow/ stripped pods (24.92 ± 5.77%).

For the correlations with the productive traits, valve weight per plant and mean valve weight increased when the shattering level increased, with the opposite for 100-seed weight and Harvest Index at pod level, which decreased when the shattering level increased (Table 6). In more detail, the oneway ANOVA between the dehiscent vs. indehiscent introgression lines showed that the former had significantly higher valve weight per plant and mean valve weight than the latter, with increases in the indehiscent introgression lines of 35.4% (*P* = 0.0085; *t*-test) and 26.2% (*P* = 0.0004). In contrast, the opposite was seen for the 100-seed weight and the Harvest Index at pod level, where the indehiscent introgression lines showed an increase of 15.9% (*P* = 0.0002) and 8.9% (*P* < 0.0001), respectively, to the dehiscent introgression lines.

The correlations between the levels of shattering with the six precision phenotyping variables that describe the pod morphology were significant (from *P* < 0.0001 to = 0.021) and all negative (Table 6).

## Discussion

### Field phenotyping of the shattering trait in common bean

High variations for both *levels* and *modes* of pod shattering were recorded. All of the shattering types were distinguishable, which varied from completely indehiscent to “twisting,” passing through the two defined “intermediate” states of “fissured” and “shattering but non-twisting” (Lamprecht, 1932). Each introgression line was characterized by counting and classifying the pods into these four categories, with the degree of resistance to manual shattering also independently measured.

As shown by the partition analysis, the best predictor of resistance to manual shattering was the *level* of shattering (percentage of shattering pods per plant), while the *mode* of shattering (twisting/non-twisting) was less relevant. Moreover, a low threshold of shattering pods per plant (10%) was sufficient to distinguish between the low and medium-high resistant introgression lines. All this suggests that shattering might be controlled by the “switching” of the mechanism of control that determines the abrupt change in the possibility of splitting the pod valves. These data also indicate that when considering both natural or artificial plant populations, genetic studies aimed at deciphering the genetic architecture of the pod shattering trait would benefit from a step-wise approach that comprises the following: (1) comparing indehiscent vs. dehiscent introgression lines (regardless of the degree of shattering); (2) considering only dehiscent introgression lines (regardless of the mode); and (3) considering separately among the dehiscent introgression lines those with twisting and non-twisting pods. Indeed, this approach would allow the genetic basis of the *occurrence* of shattering (yes/no) and also its *tuning* (low/high) and *mode* (twisting/non-twisting) to be described. It should also be noted that the variable of “fissured pods” did not prove useful to predict resistance to manual shattering; this suggests that this trait would be better investigated separately from the others.

### Element composition and cell-wall analysis of the pod valves

These shattering and non-shattering genotypes clearly differed in their carbon contents. The contents of carbon, hydrogen, and nitrogen are expected to be stoichiometrically correlated to the amount of organic matter in the tissues (Chiariello et al., 2000), and thus to the cumulative content of carbohydrate, protein, lipid, and all other organic compounds. However, in plants, differences in the carbon content have frequently been correlated to differences in lignin content (Loader et al., 2003). This was also the case for the valves of common bean; indeed, the cell-wall analysis here confirmed that the differences in the carbon contents between the shattering and non-shattering types were mainly correlated with the differences in the lignin contents, in comparison with the other cell-wall components (i.e., hemicellulose, cellulose).

There was an abrupt increase in the carbon content at a level of shattering of ~7.14%. This value was similar to the threshold of 10% of the shattering pods per plants that explained the largest proportion (65%) of the variance for resistance to manual shattering. These observations suggest that environmental effects might act on the level of shattering, and that the complementation of the whole-plant characterization and chemical element composition analysis can lead to more precise and alternative or complementary phenotyping option.

The comparison of the data in the present study with those from the literature reveal differences between common bean and soybean. Indeed, in soybean, the high-shattering cultivars were shown to have similar lignin contents (not higher, as observed here for common bean) to the low-shattering cultivars (see Table 1 of Romkaew et al., 2008). Moreover, in F2 and back-crossed populations between yardlong and wild cowpea, among these three fiber components, the contents of hemicellulose showed the highest correlation with pod shattering (Suanum et al., 2016). All this suggests that there are histological differences between other legumes species and common bean, which appear to be due to differences in the patterns of cell-wall lignification of the pod tissues, or differences in the prevalent fiber type.

### Histological characterization of the pod valves

The data from the histological characterization of pod valves in the present study parallel the observations at the chemical level. Indeed, overall, cell-wall lignification is much more pronounced in the shattering type than the non-shattering type for common bean. Specifically, the ventral sheath of the wild-like genotype (MG38) was characterized by very strong sclerenchymatization of the cells, while the opposite was seen for the domesticated cultivar (MIDAS). This observation is consistent with Prakken (1934), who indicated this anatomical difference at the basis of the presence/absence of pod strings, and on the basis of the shattering/non-shattering phenotypes. Moreover, the presence/absence of pod strings has been shown to be under the control of the *St* gene (Koinange et al., 1996).

The histological differences between the shattering and non-shattering genotypes for common bean in the present study appear to be more pronounced than those for soybean. Indeed, for soybean, the differences were limited to the dehiscent zone, where excessive lignification of the fiber cap cells was seen in the cultivated non-shattering genotypes, as compared to the wild shattering genotypes (Dong et al., 2014). Furthermore, a major gene, known as *SHA1-5*, was identified as being responsible for lignin deposition in the fiber cap cells of soybean (2014). The present study did not show any clear histological differences in the common bean dehiscence zone. Albeit it cannot be completely excluded that there were some undetected histological differences here in the dehiscence zone in common bean, these data suggest that the histological basis of pod shattering in bean and soybean are different, at least partially.

Furthermore, the shattering genotypes here had a fibrous and strongly lignified cell layer between the inner and outer parenchyma of the pod wall, while this was not seen for the non-shattering genotypes. This difference was also noted for common bean by Prakken (1934), in their comparison of the “stringy” and “stringless” types. Funatsuki et al. (2014) noted that in cultivated soybean, the differential lignification in the lignin-rich inner sclerenchyma of the pod walls influenced valve twisting and pod shattering. Furthermore, they showed that lignin deposition in this layer was under the control of a major gene, known as *PDH1*. We note here that in soybean, the difference between the shattering and non-shattering types appears to be in the *degree* of lignification of this inner sclerenchyma layer (Funatsuki et al., 2014), while the present study indicates that in common bean this difference is much stronger, with the presence/absence of the lignified layer seen. This suggests that the role of this lignified layer in the shattering might be more relevant (or at least different) in common bean compared to soybean. This might mark another difference between these two closely related crops, of common bean and soybean. Prakken (1934) suggested that in common bean, the control of the traits of “stringlessness” (which depends on the characteristics of the ventral sheaths) and “parchment” (which depends on the layer between the inner and outer parenchyma of the pod wall) was independent, and in both cases was under simple monogenic control. Another study suggested oligogenic control for the stringless trait, with the contribution of either environmental effects or epistatic interactions (Dong and Wang, 2015). Thus, in common bean, the artificial selection might have targeted multiple genes to minimize the seed loss during domestication. This evokes a scenario that arises from the joint consideration of the data obtained in soybean by the independent studies of Dong et al. (2014) and Funatsuki et al. (2014).

Based on the data in the present study, two further conclusive considerations can be made that might be useful to support the identification of shattering genes in common bean. First, it is likely that the genes underlying the shattering trait in common bean are involved in the regulation of the secondary cell-wall deposition or fiber-cell differentiation. This is well-supported by the data from the chemical analysis and the anatomical–histological investigations here; indeed, fibers are mainly composed of sclerenchymatic cells, that have well-developed secondary cell walls. This possibility is also suggested by the data of Suanum et al. (2016), who reported co-localization of QTLs for pod fiber content and pod shattering in back-cross populations between yearlong bean and wild cowpea. Secondly, as the comparison with the literature indicates some chemical and histological differences between soybean and common bean, it might be useful to consider as candidate genes not only those involved in the shattering of soybean, but also those from other phylogenetically more distant species (Dong and Wang, 2015; Li and Olsen, 2016).

### Relationships between shattering and the other plant traits

The shattering levels were very poorly correlated with the other morpho-phenological traits and productive characteristics of the plants investigated here. However, an interesting consideration arises from the observation that shattering is significantly (albeit poorly) associated with low 100-seed weight, small pod size, and low Harvest Index at pod level. This suggests that pod shattering might have an “energy cost” for the plant (McGinley and Charnov, 1988; Chiariello et al., 2000); i.e., the synthesis of the biomolecules and the creation of the tissues needed for shattering might reduce the resources available for seed and pod development. In agreement with this energy cost hypothesis, the carbon contents of the pod valves were strongly and positively correlated with the levels of shattering. This all suggests that shattering can be better viewed as a syndrome at the pod level. However, as the same data can be explained by pleiotropic effects or linkage drag, more data will need to be collected also in other species to confirm this hypothesis.

## Conclusions

Pod shattering in common bean was investigated in the present study. With this objective, we set up and adopted a pipeline for phenotypic characterization of this trait. Four main results were achieved: (1) very high shattering levels can be obtained with a high percentage of either twisting or non-twisting pods, or with a balanced combination between these two; i.e., in common bean, the *modes* of shattering do not have any great impact on the *levels* of shattering; (2) shattering appears to be controlled by a “switching” mechanism that determines an abrupt change in the ability to split the pod valves; (3) high shattering levels is correlated with high carbon and lignin contents of the pod valves, and with specific histological charaterstics of the ventral sheath and the inner sclerenchymatic layer of the pod wall; and (4) shattering appears to have a “cost.”, and it might be more exhaustively described as a “syndrome” at the pod level.

Overall, our pipeline will help with the deciphering of the genetic architecture of shattering in different crops, thus facilitating comparative studies in legumes.

## Author contributions

Designed the project: DR and RP. Managed the project: DR, GA, and RP. Wrote the article: MM and DR. Contributed to the drafting and the critical revision of the article: DR, MM, MR, GA, EBi, EBe, DA, DF, LN, TG, and RP. Contributed plant materials: EBi, EBe, LN, GA, and RP. Performed phenotypic characterization under field conditions MM, DF, TG, and DR. Histological analysis: DA, MM, and DR. Collect data from element composition and cell-wall analyses: MM. Analyzed and interpreted data: DR, MM, MR, RP, and GA. Edited the article: DR, MM, MR, EBi, EBe, GA, and RP. All of the author approve the final version of the manuscript.

### Conflict of interest statement

The authors declare that the research was conducted in the absence of any commercial or financial relationships that could be construed as a potential conflict of interest.
