# Supplementary material for: A Comprehensive Phenotypic Investigation of the “Pod-Shattering Syndrome” in Common Bean
Source: Front Plant Sci. 2017 Mar 3;8:251. doi: 10.3389/fpls.2017.00251 (PMC5334323; doi:10.3389/fpls.2017.00251)
Supplement: Supplementary file 1 [file Presentation1.PDF]

A comprehensive phenotypic investigation of the 'pod-shattering syndrome' in common bean

Supplementary materials

2. **Materials and methods**

2.2. **Phenotypic characterization**

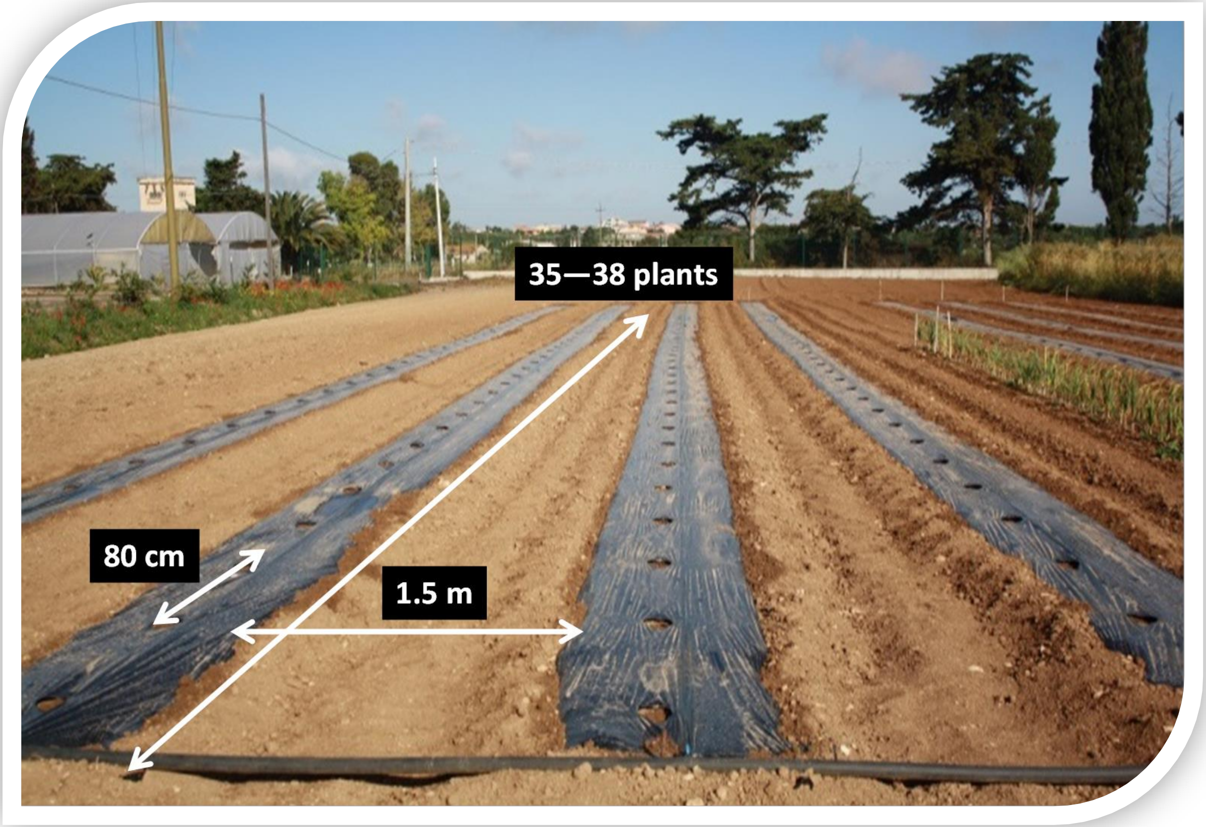

Supplementary Figure 1. Field trial design.

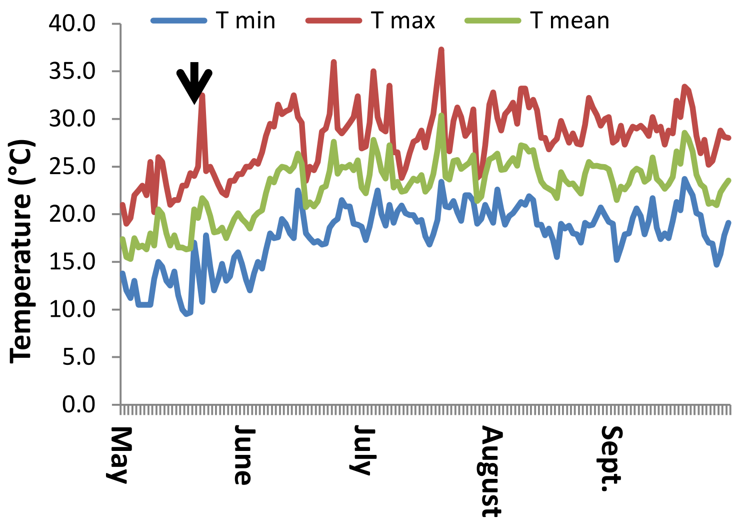

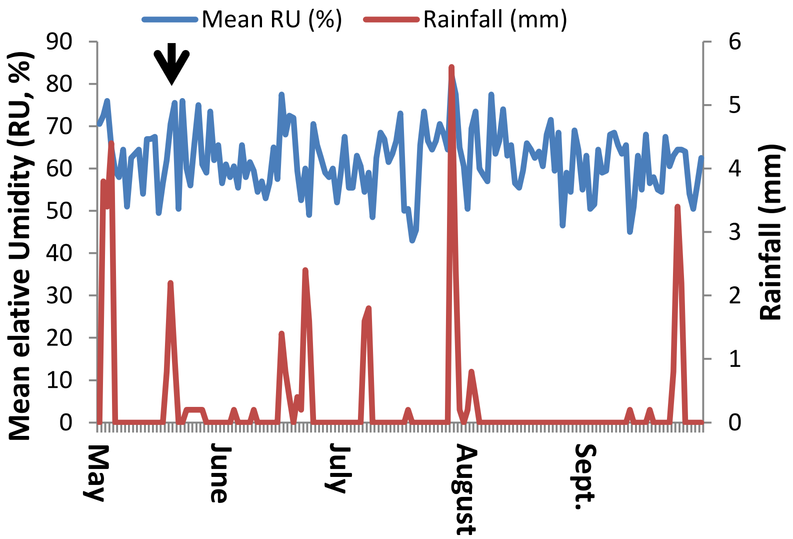

Supplementary Figure 1b. Meteorological conditions during field trial. Temperature (minimum, average, maximum; °C), average relative (umidity, RU%) and rainfall (mm) are reported for the period May-September. The black arrow indicates sowing date.

2.2.1. **Measuring pod shattering in the field**

The scale from 1 to 9 for the pod shattering measurements was defined as follow:
1: Lower than MG38 (coded very low in Figure 2C).
2: Equal to MG38 (low).
3: Higher than MG 38 but closer to MG38 than to the intermediate type (low-medium).
4: Higher than MG38 but closer to intermediate type than to MG38 (medium-low).
5: Intermediate between MIDAS and MG38 (medium).
6. Lower than MIDAS, but closer to the intermediate type than to MIDAS (medium-high).
7: Lower than MIDAS but closer to MIDAS than the intermediate type (high-medium).
8: Equal to MIDAS (high).
9: Higher than MIDAS (very high).

Supplementary videos:
1. High resistance to manual shattering (score, 8)
2. Very low resistance to manual shattering (score, 1).

2.2.3. **Cell-wall analysis**

To obtain the neutral detergent fiber (NDF), for each sample, 0.50 g pulverized tissue was used and placed into filter bags. The bags were put into the analyser (ANKOM220 Fiber Analyzer). This instrument performs digestion at 100 °C ±0.5 °C at a pressure between 10 psi and 25 psi. After adding 2 L neutral detergent solution, the samples were agitated in this solution at 100 °C for 80 min. The neutral solution was composed of sodium lauryl sulphate and EDTA, with pH ~7.0. This allows the removal of the soluble fractions present in the plant cells (i.e., pectins, sugars, starch, proteins, lipids), and retains the insoluble vegetable fraction of the cell wall. After agitation, the samples were washed twice with hot water and twice with deionized water, dried at 100 °C, and weighed. They were finally incinerated at 500 °C. The NDF is expressed as percentages of dried organic matter after subtracting the weight of the ashes. The NDF thus represents the total content in the cell wall of the analyzed sample. To obtain the acid detergent fiber (ADF), for each sample, 0.50 g pulverized tissue was used, which was placed in another filter bag to be analysed in the analyser (ANKOM220 Fiber Analyser). In this case, 2 L acid detergent solution were added (20 g cetyl trimethylammonium bromide added to 1 liter of 1 N H_2SO_4). The samples were agitated at 100 °C for 60 min. After this, the samples were washed twice with hot water and twice with deionized water, dried at 100 °C, and weighed. The ADF is expressed as percentages of dried organic matter. The ADF mainly represents an intermediate step that is necessary to obtain the acid detergent lignin (ADL; see below). However, the difference of NDF minus ADF roughly estimates the content of hemicellulose of the analyzed samples (**Van Soest and Wine, 1967**). To obtain the ADL, the ADF was treated with 72% sulfuric acid for 3 h, with mixing every 30 min. The samples were washed twice in hot water, twice with deionized water, dried at 100 °C, weighed, and finally incinerated at 500 °C. The ADL is expressed as percentages of dried organic matter after subtracting the weight of the ashes. The ADL is an estimation of the lignin content of the analyzed samples. Moreover, the difference in terms of ADF minus ADL represents an estimation of the cellulose content of the analyzed samples (**Van Soest and Wine, 1967**).

2.2.4. **Anatomical and histological study of pod valves**

1% toluidine blue O staining: 1 g toluidine blue O in 100 mL water.

Cochineal carmine and iodine green staining: The sections were first treated for 2 min with green iodine, which was acidified before use. The sections were then washed several times with 70% ethanol, and finally with water. Then the sections were treated with cochineal carmine for 30 min. There followed three washes of 10 min each with 75% ethanol, 95% ethanol and 100% ethanol, in series. The sections are then subjected to treatment with xylene for 10 min.

Paraffin embedding: To allow the paraffin to penetrate into the plant tissues, they were first dehydrated through three treatments with ethanol (50%, 75%, 100%, in series). The ethanol was then replaced by an organic solvent (BioClear, xylene) with three treatments (75% ethanol and 25% BioClear, 50% Ethanol and 50% BioClear, 25% ethanol and 25% BioClear), each of 30 min. The tissues were then treated with BioClear overnight. The BioClear was gradually replaced by paraffin with three treatments (75% BioClear and 25% liquid paraffin, 50% BioClear and 50% liquid paraffin, 25% BioClear and 75% liquid paraffin), each of 1 h, finishing off with 100% paraffin for 1 h. The samples were then embedded in the paraffin overnight. The paraffin-embedded tissues were then cut, and 10-µm sections were obtained using a sliding microtome (Reicher). The paraffin was then removed with four treatments with ethanol (100% ethanol, 95% ethanol, 75% ethanol, 50% ethanol, in series), with a final wash with water.

2.2.5 **Phenotyping other plant characteristics**

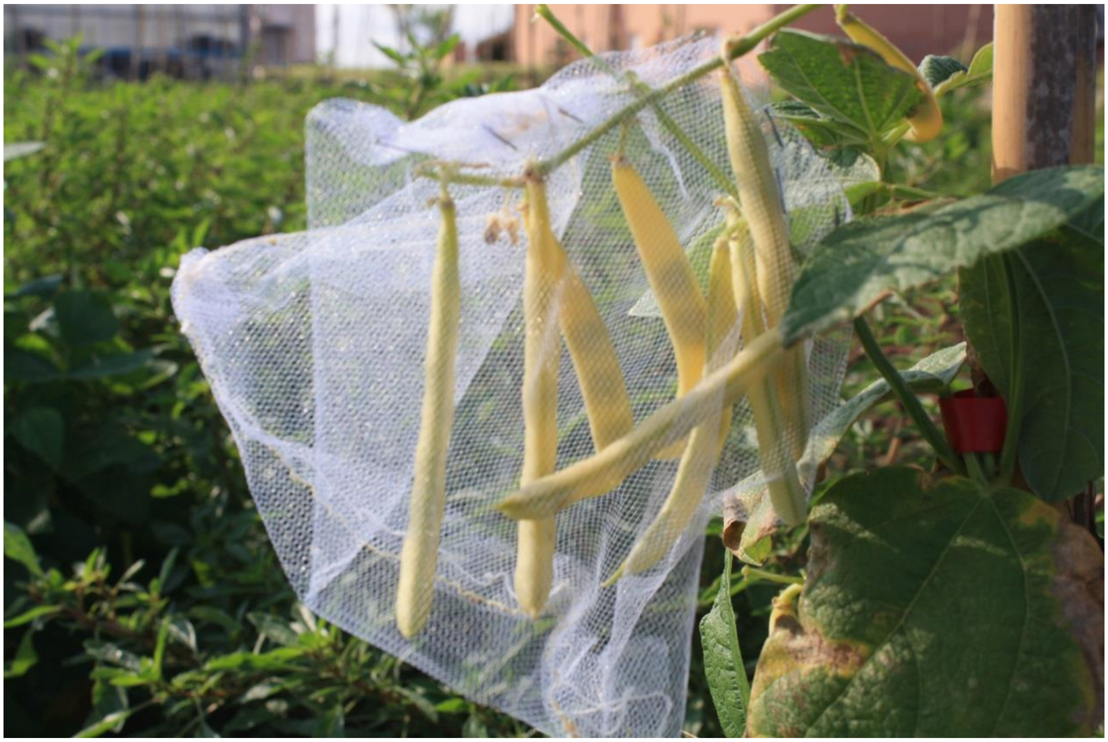

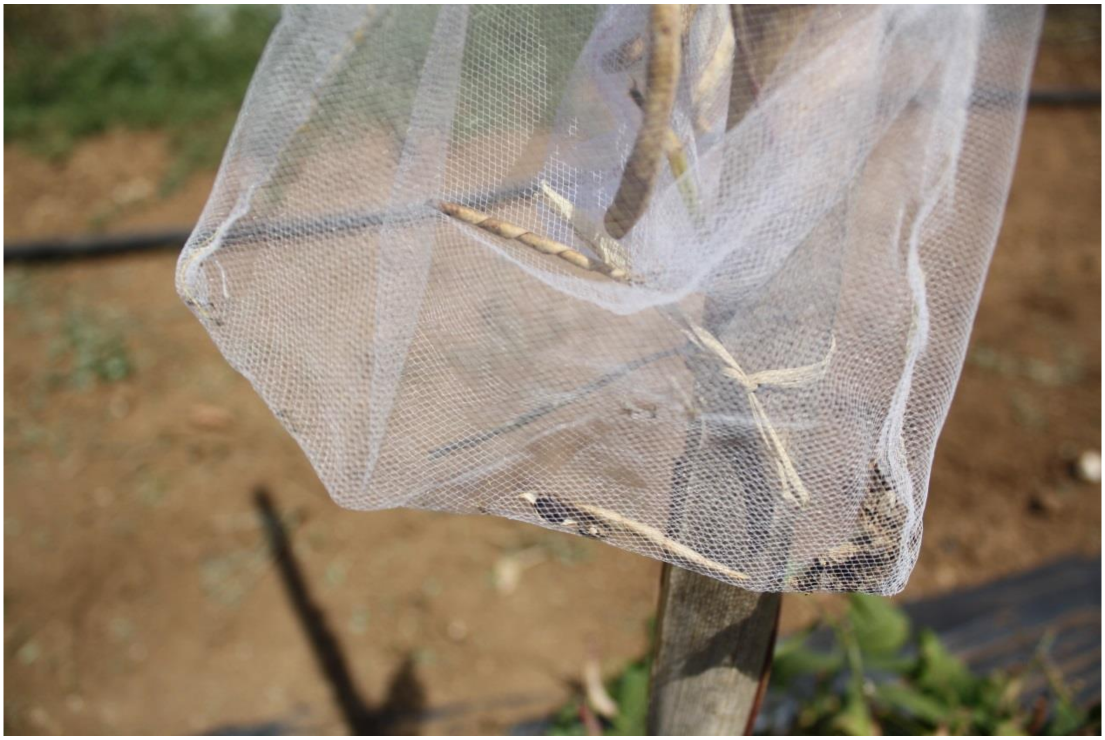

Supplementary Figure 2. Bean pods loosely wrapped with plastic netting to avoid loss of seeds following dehiscence.

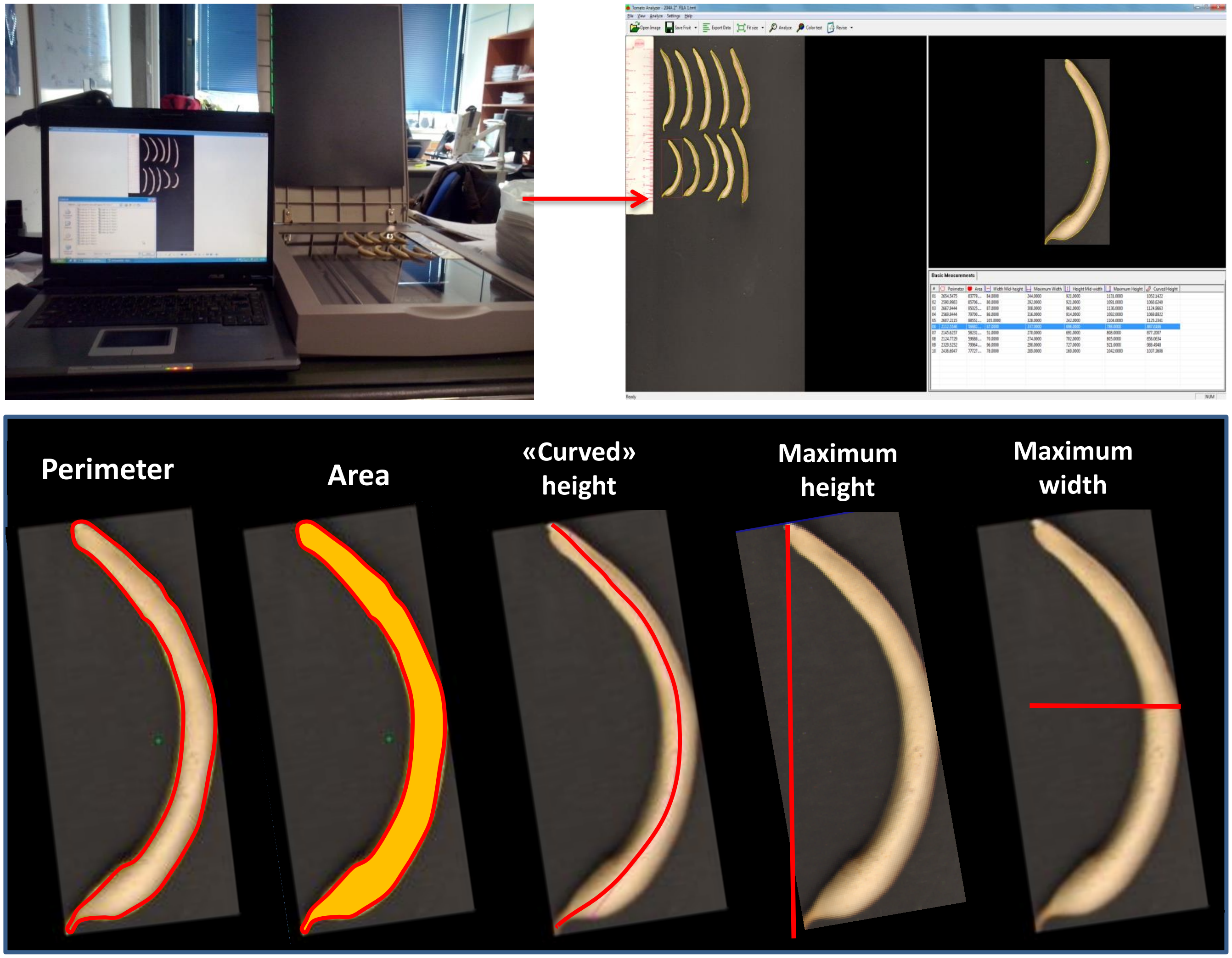

Supplementary Figure 3. Representative photographs of steps for pod precision phenotyping. Scans were obtained for 10 pods per bean line, with automatic determination of the five pod traits illustrated using the Tomato Analyzer software.

3. Results

3.1 **Shattering *level* and *mode***

Supplementary Table 1 – Results of ANOVA: < 7.14% or ≥ 7.14% of shattered pods vs ILs families, all ILs.

**All families with >2 ILs**			
Trait	R^2 (R^2_{adj})	$F_{101,137}$	P
< or ≥ 7.14% of shattered pods	0.98 (0.96)	75,46	$<10^{-4}$

We determined among-family phenotypic structure for the shattering trait.
We applied one-way ANOVA considering family as factor and shattering ability as independent variable. Results are presented in **Supplementary Table** 1. Results showed that within families there was very high homogeneity for shattering ability and the variance was almost all *between* families of ILs.

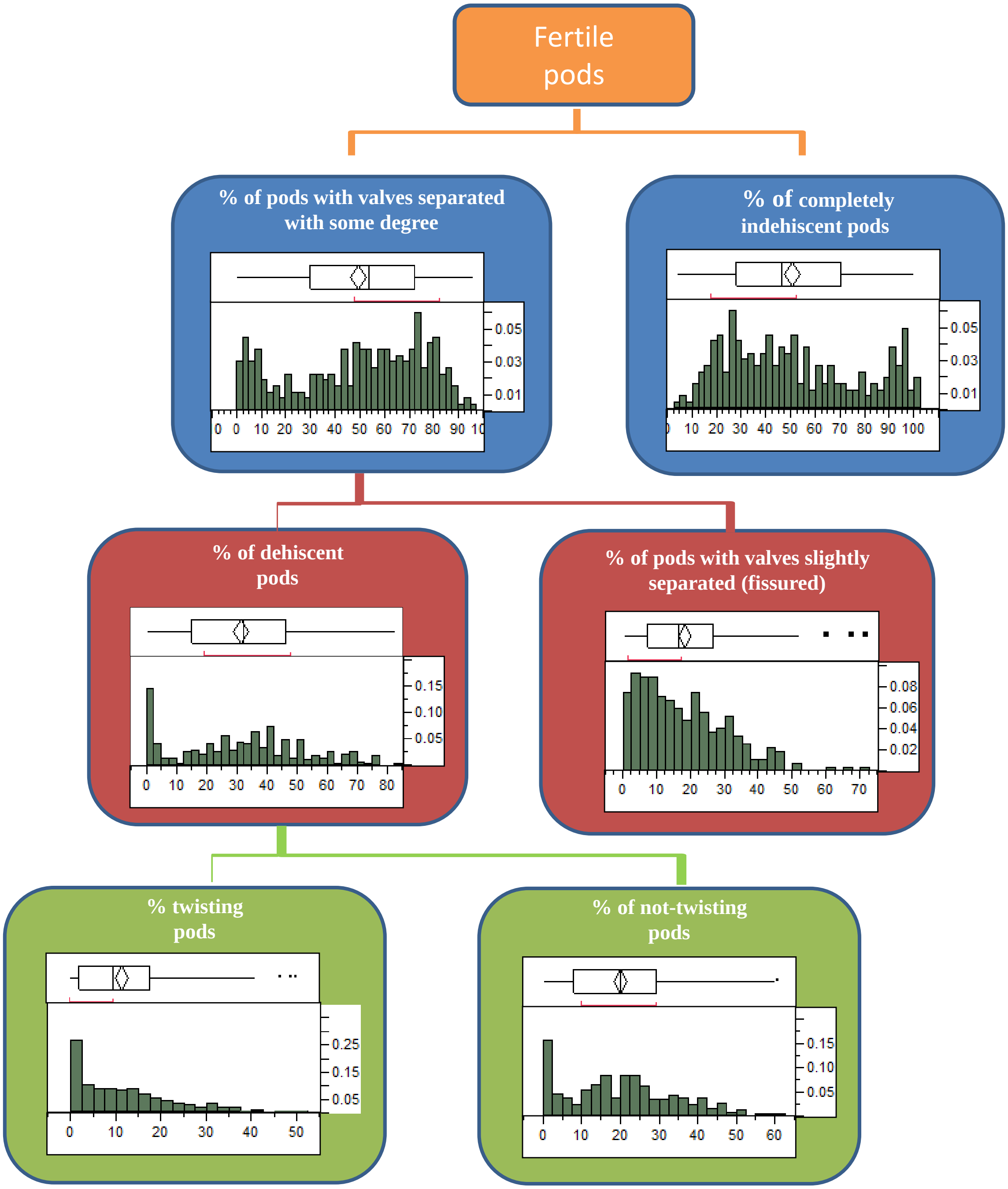

Supplementary Figure 4. Frequency distributions of the variables used to measure shattering in the analyzed population of common bean. All of the variables were expressed as percentages of the fertile pods. Sample size, n = 267, for all of the distributions.

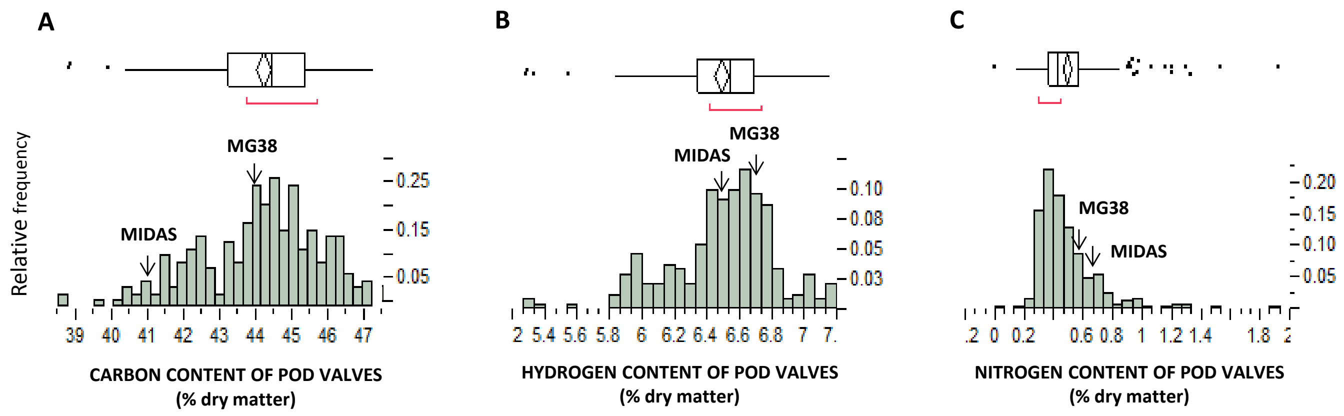

Supplementary Figure 5. Frequency distributions for carbon, hydrogen and nitrogen contents (precentages dry weight) within the population of bean introgression lines.

3.4. **Correlations between element compositions and cell-wall analyses**

Supplementary Table 2. Results of the stepwise multiple regression (as forward selection) considering the carbon content as the dependent variable, and the lignin, hemicellulose, and cellulose as the independent variables.

**Parameter**	**Statistic**			
	**d.f.**	**S.S.**	**F**	**P**
Intercept	1	0	0	1
Hemicellulose (NDF-ADF)	1	2.63	2.60	0.118
Cellulose (ADF-ADL)	1	3.25	3.29	0.081
Lignin (ADL)	1	28.31	26.51	1.85×10^{-5}

d.f., degree of freedom; S.S. sum of squares; F = F ratio; P = significance level

3.5. **Anatomical and histological analysis of pod valves**

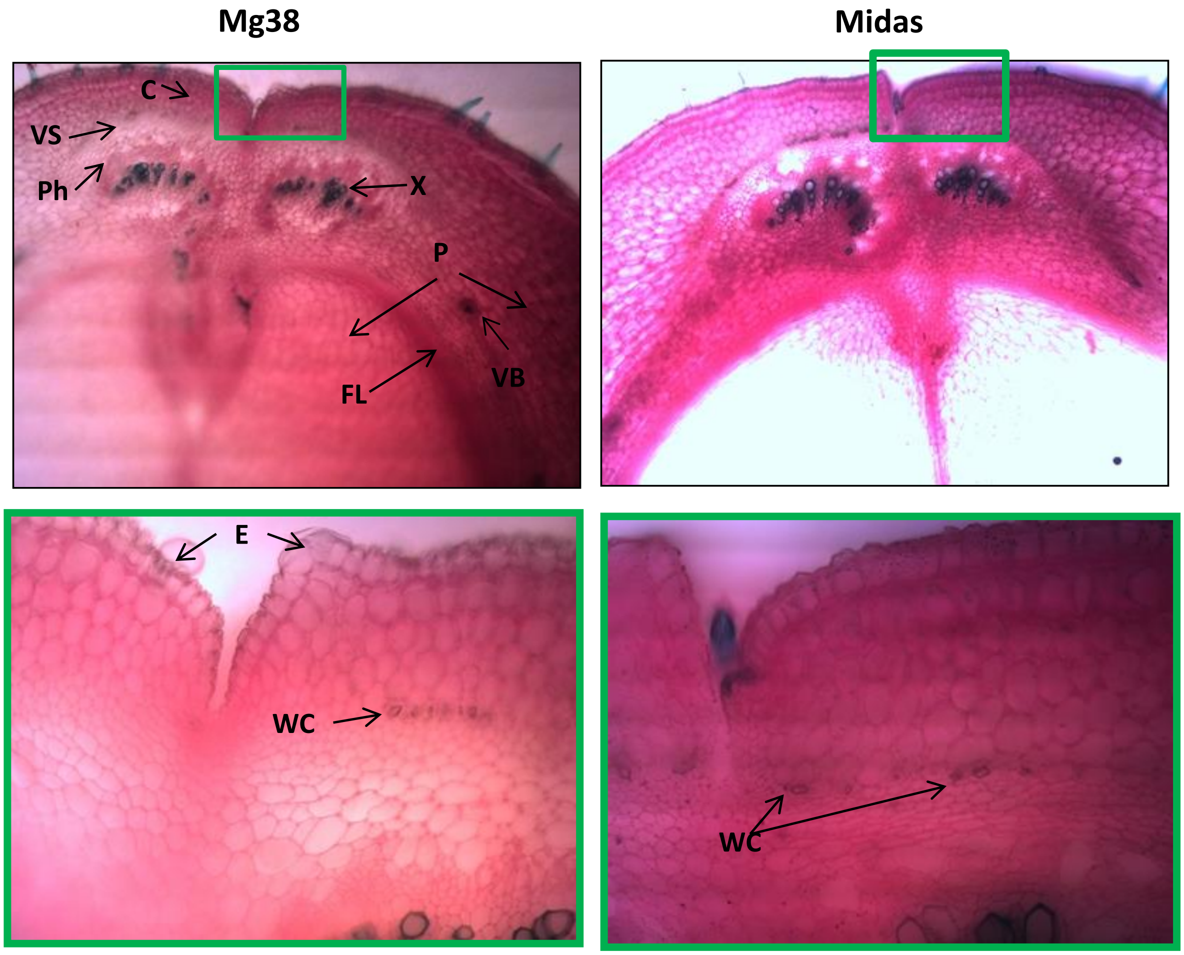

Supplementary Figure 6. Representative images from the histological study of the ventral sheath of the pod valves of 5-day-old pods. C, collenchyma; E, epidermis; FL, fibrous layer; P, parenchyma; Ph, phloem; VB, vascular bundle; VS, ventral sheath; WC, wood cells; X, xylem.

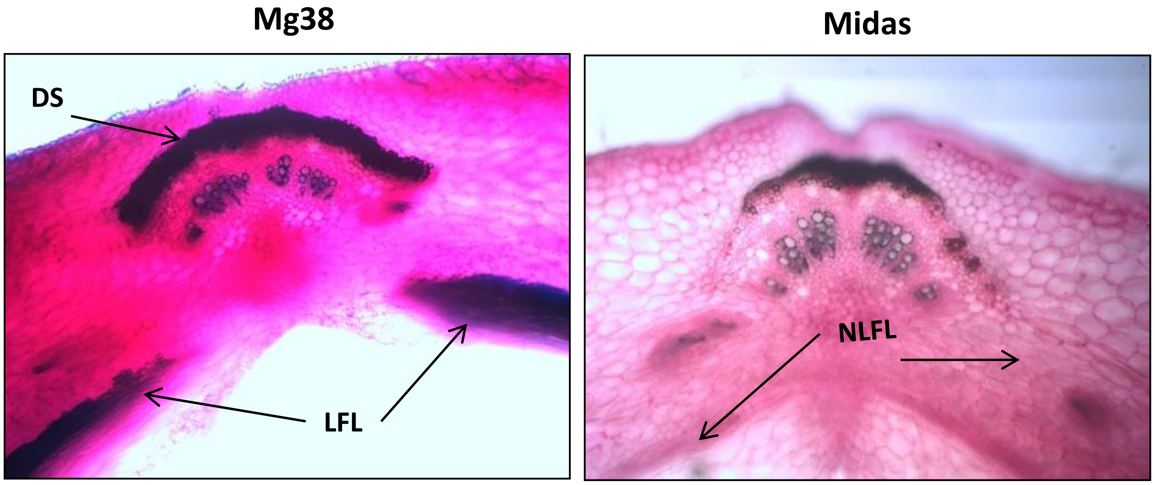

Supplementary Figure 7. Representative images of the dorsal sheath of the highly dehiscent MG38 (left) and the indehiscent MIDAS (right). DS, dorsal sheath; LFL, lignified fibrous layer; NLFL, nonlignified fibrous layer.

(A)

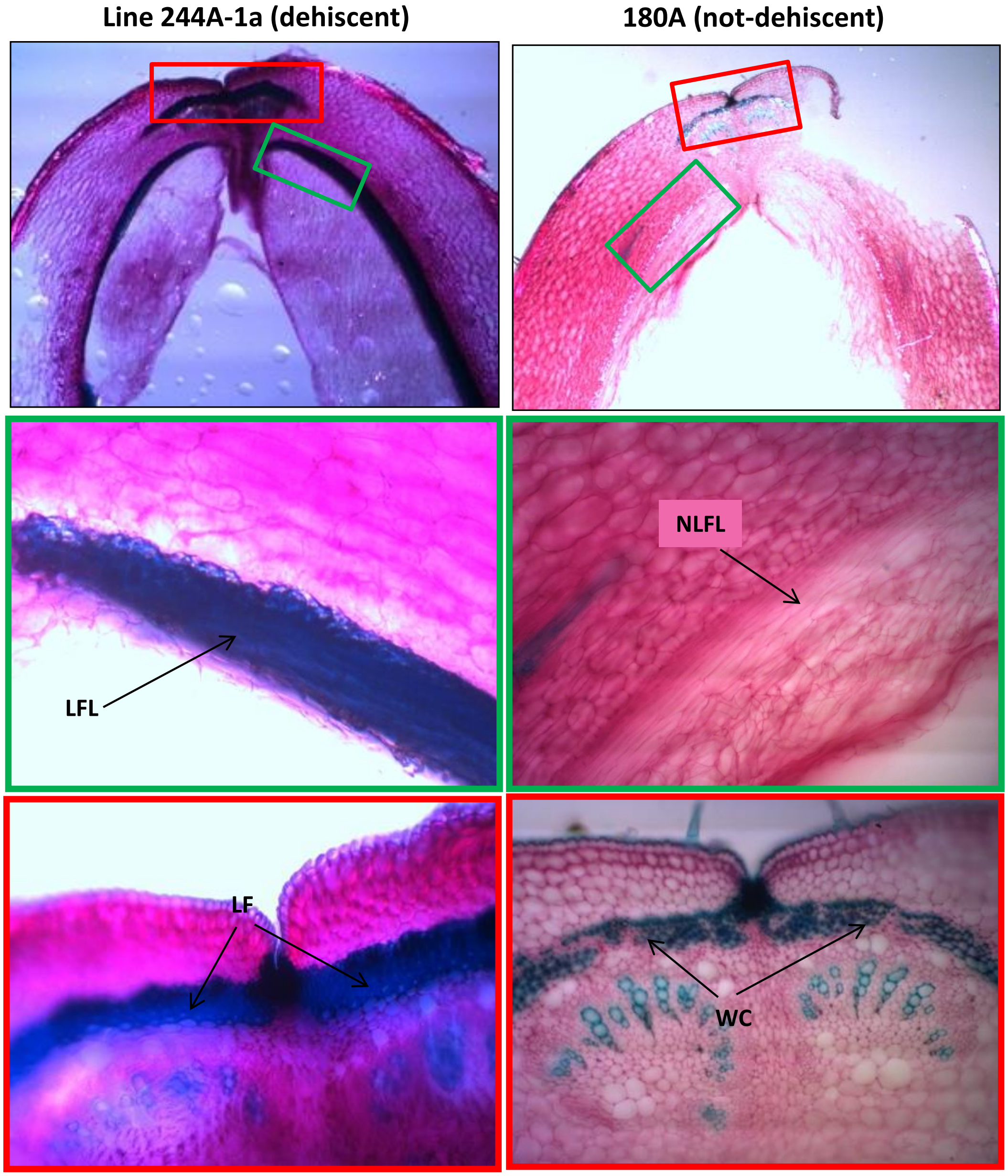

(B)

244A-1a (higly dehiscent)

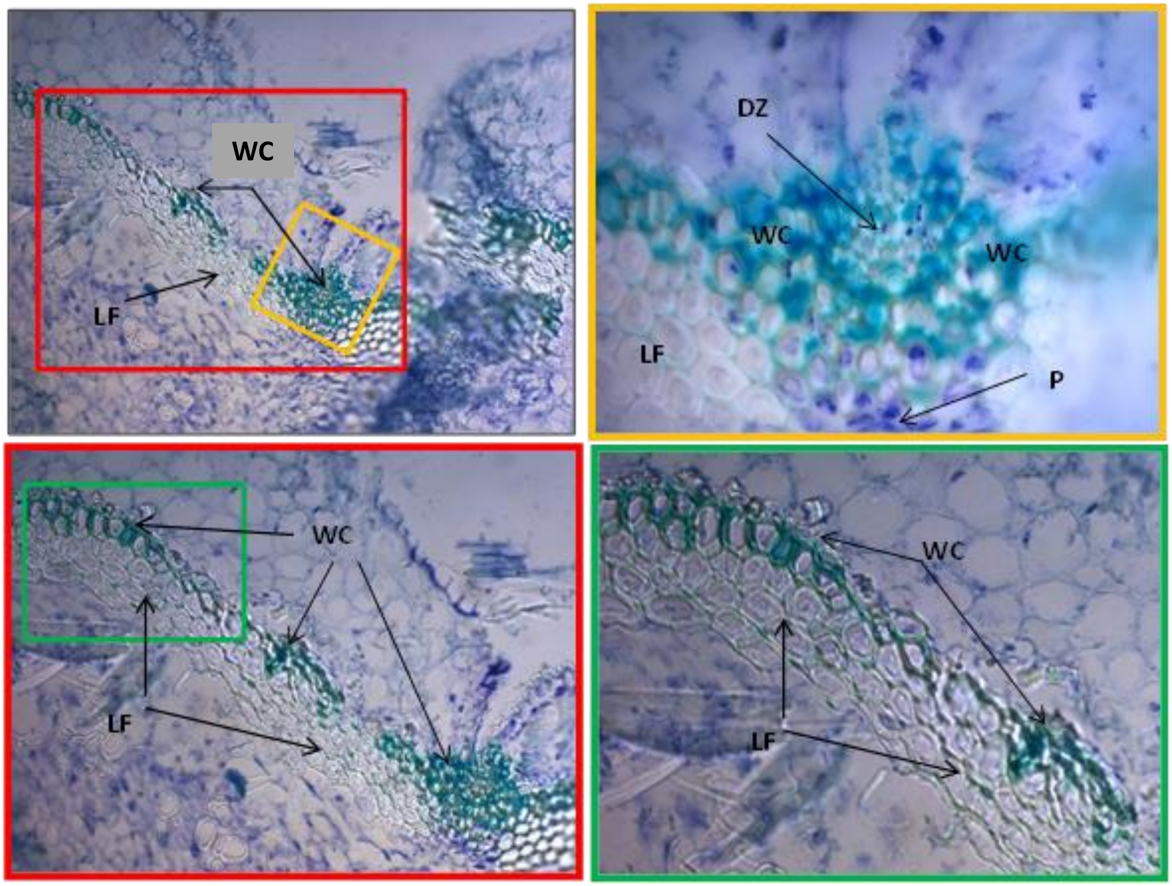

(C)

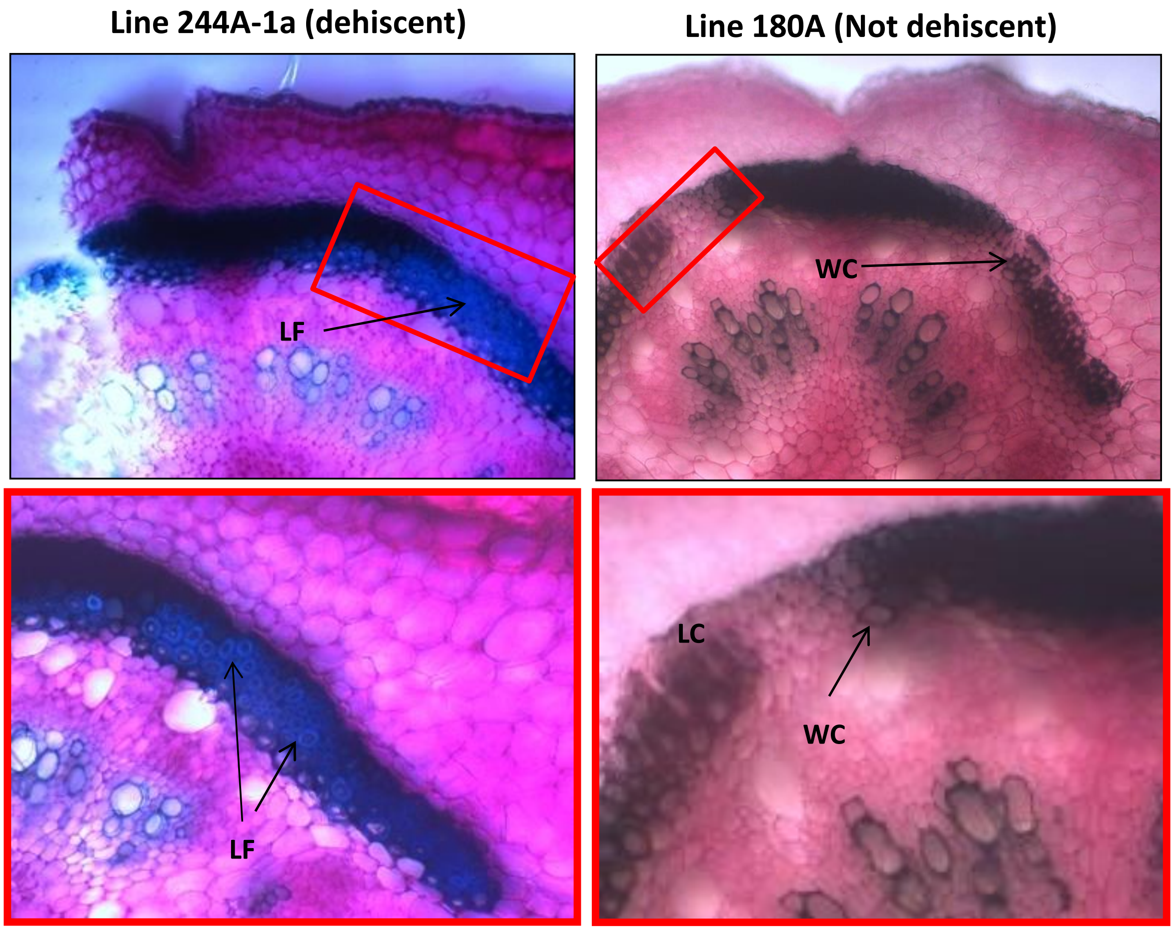

Supplementary Figure 8. Representative images of ventral and dorsal sheaths of two specific introgression lines. (A) Comparison of the ventral sheath between two highly contrasting introgression lines, 244A-1a (highly dehiscent) and 180A (indehiscent) as with iodine-green staining. (B) Particular of the ventral sheath of the dehiscent line 244A-1a, as TBO staining following paraffin embedding. (C) Comparison of the dorsal sheath between 244A-1a (highly dehiscent) and 180A (indehiscent) as by iodine-green staining. LFL, lignified fiber layer; NLFL, nonlignified fiber layer; LF, lignified fibers; WC, wood cells; DZ, dehiscence zone; P, parenchyma.

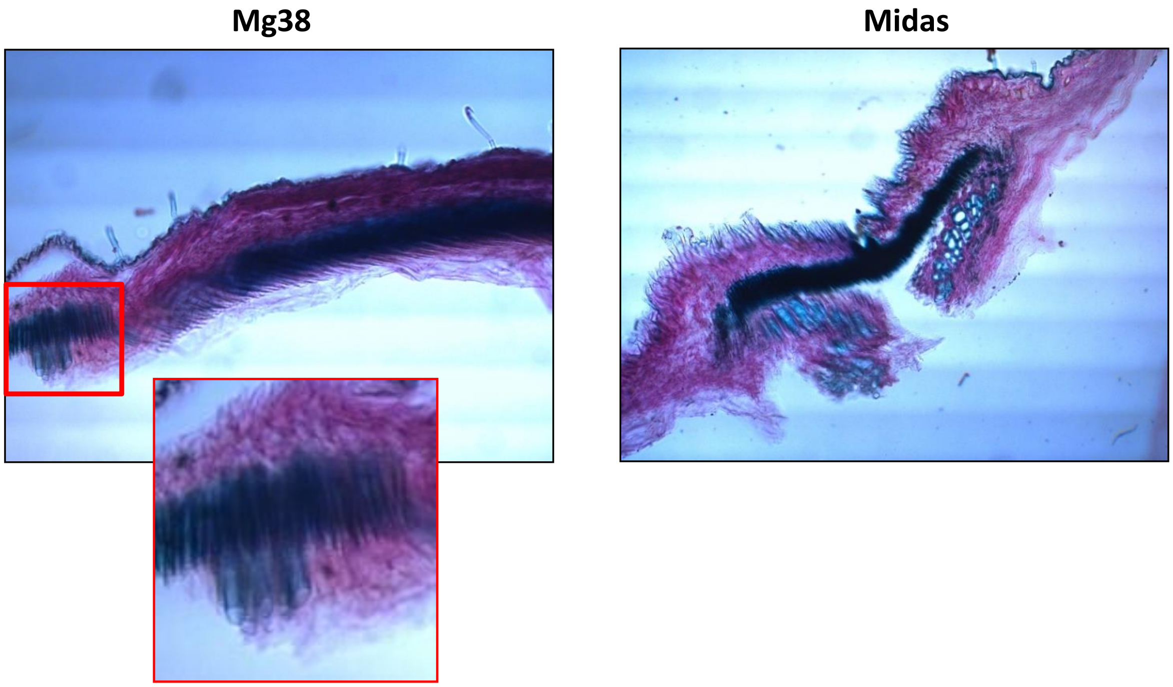

Supplementary Figure 9. Representative images for the comparison between MG38 (dehiscent) and MIDAS (indehiscent) for the ventral sheath at the maturation stage. Magnified inset: Detail from red box indicated fibers.
